# Dietary Restriction and Rapamycin Affect Brain Aging in Mice by Attenuating Age-Related DNA Methylation Changes

**DOI:** 10.3390/genes13040699

**Published:** 2022-04-15

**Authors:** Zhilei Yin, Xinpeng Guo, Yang Qi, Pu Li, Shujun Liang, Xiangru Xu, Xuequn Shang

**Affiliations:** 1 School of Computer Science, Northwestern Polytechnical University, Xi’an 710060, China; yinzhilei@zzuli.edu.cn (Z.Y.); 2017100647@mail.nwpu.edu.cn (X.G.); yang.qi@mail.nwpu.edu.cn (Y.Q.); 2 College of Software Engineering, Zhengzhou University of Light Industry, Zhengzhou 450001, China; lipu@zzuli.edu.cn (P.L.); liangsj@zzuli.edu.cn (S.L.); 3 Department of Anesthesiology, Yale University School of Medicine, New Haven, CT 06510, USA

**Keywords:** dietary restriction, rapamycin treatment, DNA methylation, aging, hippocampus

## Abstract

The fact that dietary restriction (DR) and long-term rapamycin treatment (RALL) can ameliorate the aging process has been reported by many researchers. As the interface between external and genetic factors, epigenetic modification such as DNA methylation may have latent effects on the aging rate at the molecular level. To understand the mechanism behind the impacts of dietary restriction and rapamycin on aging, DNA methylation and gene expression changes were measured in the hippocampi of different-aged mice. Examining the single-base resolution of DNA methylation, we discovered that both dietary restriction and rapamycin treatment can maintain DNA methylation in a younger state compared to normal-aged mice. Through functional enrichment analysis of genes in which DNA methylation or gene expression can be affected by DR/RALL, we found that DR/RALL may retard aging through a relationship in which DNA methylation and gene expression work together not only in the same gene but also in the same biological process. This study is instructive for understanding the maintenance of DNA methylation by DR/RALL in the aging process, as well as the role of DR and RALL in the amelioration of aging.

## 1. Introduction

The aging process is influenced by both external factors and inherent genetic factors. The retardation of aging at the molecular level may underlie the mitigation of aging at the functional level. A growing number of studies have utilized the remodeling of DNA methylation profiles to confirm that types of environmental exposure such as dietary restriction (DR) and rapamycin treatment lasting long-term (RALL) can ameliorate aging in multiple species [1,2,3]. Epigenetic modification is regarded as the interface between environmental factors and genetic inheritance. Many investigations of aging have been performed on both model species and humans, although the mechanism of epigenetic modification and aging is far from clear [4,5,6,7].

DNA methylation plays an important role in development and the aging process [3,8,9,10]. Li, Y. et al. mentioned that aging causes a decrease in global DNA methylation, while the promoters of some genes switch from unmethylated to methylated [3]. Researchers have proposed aging clock models using DNA methylation in multiple species [1,11,12,13,14,15]. The variation tendencies of DNA methylation with aging and the epigenetic biomarkers in disease have been extensively studied [16]. For example, Yan, H. et al. found that different methylation CpG was contained in the promoter region of 169 genes in 268 colorectal cancer patients [17]. The role of DNA methylation and its tendency with age, however, have been distinguished in multiple tissues such as liver [2,18], blood [19], and heart [20]. The variation of DNA methylation in the brain plays an important role in cognitive decline, which is a significant manifestation of aging [19,21,22,23]. The hippocampus is one of most crucial regions in the brain and is closely associated with the cerebral cortex for learning, memory, and cognitive behavior. During the normal aging process, humans and animals experience age-related memory and cognitive impairments [24,25]. The functional decline of hippocampal neurons with age is the key alteration, which likely results from defects in neuronal plasticity [26]. We and others had collectively discovered that altered synaptic plasticity gene expression in the hippocampus and frontal cortex neuronal cells/tissues with advancing age and age-related neurodegenerative diseases, although the molecular mechanisms underlying this altered gene expression are largely unknown [27,28]. Epigenetics has emerged as a possible mechanism controlling gene expression and a potential causative factor of brain aging and age-related neurodegenerative disorders such as Alzheimer’s disease [29,30]. DNA cytosine methylation is a major epigenetic mechanism in higher eukaryotes, including plants, rodents, and humans, and accumulated studies suggest that impairment of DNA methylation plays a pivotal role in regulating mouse brain aging and neurodegeneration [31,32]. However, the mechanism connecting DNA methylation and brain aging remains unclear [2,3,4,23,33]. Another question is how DR and RALL affect DNA methylation drift along with aging. It is therefore important to explore the regulating effect of DNA methylation on brain aging and aging-related diseases.

Several studies have reported that DR and RALL can remodel the DNA methylation profile to allay the adverse effects of aging [2,34]. Oliver Hahn et al., for example, confirmed that DR can accelerate lipid metabolism efficiency by improving the DNA methylation levels of specific genes [2]. As the “mimic” of DR, RALL can also ameliorate aging [35,36,37,38]. An, J. Y. et al. demonstrate that short-term treatment with rapamycin rejuvenates the aged oral cavities of elderly mice [39]. Previous studies have reported that rapamycin can slow aging and extend lifespans in multiple species [40,41,42]. Harrison, D. E. et al. reported that rapamycin extends the median and maximal lifespans of mice when fed beginning at 600 days of age [42]. Rapamycin can also play a role similar to that of dietary restriction in multiple tissues including the liver, blood, and central nervous system [43,44,45]. Limited by DNA tissue-specific methylation features and the inherent complexity of gene expression regulation, however, previous studies have failed to agree on the precise role of DR/RALL in brain aging [46,47,48]. There are even discrepancies in the variation tendency of DNA methylation with brain aging [2].

In this study, the DNA methylation and matched gene expression levels were sequenced for each sample. To explore the effects of DR and RALL on the aging process, we focused on the differences of DNA methylation and gene expression between normal aging and aging controlled by DR or RALL. We identified DR/RALL-ameliorated genes and examined the underlying biology of those genes. As a result, we made a reasonable conjecture of the DR/RALL effects on the aging process. This study is useful for understanding not only the maintenance of DNA methylation by DR/RALL in the aging process, but also the role of DR and RALL in the amelioration of aging.

## 2. Materials and Methods

### 2.1. Study Animals and Library Preparation

To study the effects of dietary restriction and/or rapamycin on DNA methylation and gene expression in mouse aging, we studied 15 mice of different ages with or without dietary restriction and rapamycin treatment, with 3 mice in each group. BALB/C female mice were produced and maintained at the Jackson Laboratory (Bar Harbor, ME, USA). At weaning (3 weeks of age), they were housed four per box in weaning cages. After weaning, three mice in each weaning cage were assigned one of three feeding regimens using Purina LabDiet’s 5LG6 irradiated formulation of the NIH-31 (4% fat) diet. Groups of Ad libitum (AL)-fed mice, both young (3 months) and old (22 months) were given uninhibited access to grain and their intake was adjusted once every 2 weeks. A group of DR mice were fed with a 70% feeding rate of the AL mice in the same age after weaning and last lifelong (22 months of age). For the group of rapamycin-treated (RALL) mice, the rapamycin was encapsulated and 2.24 mg of rapamycin per kg body weight/day was taken with food (equivalent to AL feeding) for 3 months starting when the animals were 19 months of age [42]. The dietary-restricted mice treated with rapamycin are named RDRL (Restricted Dietary and Rapamycin treatment for a Long time) mice. That is to say, the mice of the RDRL group was subjected to both DR and RALL. All procedures were carried out and approved by Yale’s Institutional Animal Care and Use Committee (IACUC) (license number: 2013-11131), and staff veterinarians monitored the mice on a regular basis, finding no pathogens.

Mice were sacrificed by cervical dislocation according to the approved protocol and demonstrated in our previous work [33]. Then the health status of the mice was first checked anatomically. The subregions of brains were dissected out and snap-frozen in liquid nitrogen, ground into powder with a mortar and pestle in liquid nitrogen, and then stored at −80 °C.

DNA/RNA of mouse hippocampal tissues was processed as we previously described [49]. DNA/RNA isolation was carried out using the Qiagen DNA/RNA extraction kit (Qiagen, Inc., Valencia, CA, USA). In short, frozen tissues were cut into small pieces and allowed to thaw in the RLT lysis buffer with β-meta-ethanol. This was followed by disruption using a mechanical homogenizer with the appropriate speed. The tissue samples were then centrifuged; the supernatant transferred to the Qiagen DNA-binding columns. Next, 70% alcohol was added to the supernatant and transferred to the RNA-binding columns. After centrifugation, both DNA- and RNA-binding columns were washed several times and the bound DNA/RNA was eluted using Tris-HCL buffer and RNAse-free water. The DNA/RNA quality and quantity were checked using an Agrose gel system and an Agilent 2100 bio-analyzer. Only DNAs/RNAs meeting the following quality criteria (A260/A280 > 1.8, A230/A260 > 1.8, and RNA integrity number > 7) were applied for further analysis.

Sequencing was conducted commercially in HudsonAlpha Genomic Services Lab (Huntsville, AL, USA). Briefly, 100 ng of total RNA was used to prepare amplified cDNA using an Ovation RNA-Seq, a commercially available kit optimized for RNA sequencing (NuGEN Technologies, San Carlos, CA, USA). The produced double-stranded cDNA was subsequently used as the input to the Illumina library preparation protocol starting with the standard end-repair step. The end-repaired DNA with a single ‘A’-base overhang is ligated to the adaptors in a standard ligation reaction using T4 DNA ligase and 2 μM–4 μM final adaptor concentration, depending on the DNA yield following purification after the addition of the ‘A’-base. Following ligation, the samples were purified and subjected to size selection via gel electrophoresis to isolate 350 bp fragments for ligation-mediated PCR (LM-PCR). Twelve cycles of LM-PCR were used to amplify the ligated material in preparation for cluster generation. The prepared cDNA library was sequenced for 100 bp single-end reads on three flow cell lanes using the Hiseq 2000 platform. The image analysis, base calling, and quality score calibration were processed using the Illumina Pipeline Software v1.4.1 according to the manufacturer’s instructions.

Reduced representation bisulfite sequencing (RRBS) was performed as described. Briefly, 200 ng genomic DNA was digested with the methylation insensitive restriction enzyme MspI (NEB). Ends of each restriction fragment were filled in and a 3′ adenosine was added with Klenow Fragment (3′ → 5′ exo-minus; NEB). Methylated single-end Illumina adapters were ligated to the ends of the DNA fragments using T4 DNA Ligase (NEB). Fragments between 105 bp and 185 bp were purified by agarose gel extraction. The purified fragments were treated with sodium bisulfite and then amplified by PCR with long-range PCR conditions and Platinum Taq Polymerase (Invitrogen, Waltham, MA, USA). The final PCR products were sequenced on Illumina Hi-seq 2000. All of the sequence data that is presented is of high quality, with average quality scores of more than 25 for each cycle.

### 2.2. DNA Methylation Data Analysis

The *.fastq files were aligned to the mouse genome (GRCm38) using BS-Seeker2 (v 2.1.1, max-mismatch = 4) [50] and bowtie2 (v 2.0.0, default parameters) [51]. The methylation levels of the CpG sites were calculated from the *.bam files generated by BS-Seeker2. The read number and methylation level of each CpG site were included in the result files. We filtered out the non-CpG sites and CpG sites with read numbers < 5. We then divided all sequenced CpG sites into scattered bins. The maximum distance between two adjacent sites was no more than 1 kbp in each bin. The methylation level of each bin was the average methylation level of all sites in that bin. The details of DNA methylation data processing can be seen in Supplementary File S1. We computed the methylation difference of bins between the two groups using the limma package (v 3.34.9) [52]. Bins with *p*-values < 0.05 and average methylation differences > 0.1 were identified as significant different methylation regions (DMRs).

To compare the methylation variations between normal aging and aging controlled by DR or RALL, scatterplot comparison of bin-wise differences between normal age-related changes and DR/RALL-related changes. We calculated the mean methylation level of all bins in each group. Differences in methylation levels between the old and young groups were used as baseline, represented on the X-axis. The Y-axis represents the amount of change caused by the aging process under DR/RALL conditions. To obtain the global trend of methylation, we trained the coordinates of all bins by using the linear regression [53]. This analytical method can be found in reference [2]. Every point in scatterplot represents a bin and each coordinate position represents the methylation difference between two different groups. We performed linear regression analysis on all bins using the package sklearn (v 0.19.2) [53]. The Pearson correlation coefficients (PCC) were computed for all bins in each diagram in order to inspect the correlations of the bins.

Bins with significantly lower methylation levels in the normal old group than the young group were labeled aging-related hypo DMRs, while DMRs with significantly higher methylation levels were labeled aging-related hyper DMRs. DR-ameliorated DMRs were selected from aging-related DMRs in which DR could reduce the methylation status changes, i.e., bins in aging-related hyperDMRs were selected if the methylation was lower in the DR group than in the normal old group, while bins in aging-related hypo DMRs were selected if the methylation was higher in the DR group than in the normal old group. RALL-ameliorated DMRs were chosen using the same selection process as the DR-ameliorated DMRs. The gene data were downloaded from the UCSC knownGene table of GRCm38/mm10. The genes included in the DMRs of the promoter and gene body were designated different methylation genes (DMGs).

### 2.3. Gene Expression Data Processing

The *.fastq files were aligned using Tophat (v 2.1.1, default parameter) [54] to generate the *.bam file and the reference genome (GRCm38) downloaded from UCSC. The *.bam files were then processed using Cufflinks (v 2.2.1) to obtain the expression level of each gene. The FPKM values of the genes were used in further analysis. The different expression genes between the two groups were identified using Cuffdiff and the genes with *p*-values < 0.05 were selected as different expression genes (DEGs). The details of gene expression data processing can be seen in Supplementary File S1. DEGs list and the FDR-adjusted *p*-value of the test statistic can be seen in Table S1. DEGs were divided into two categories, depending on whether the expression level increases or decreases with age. DEGs with a higher expression level in old mice than in young mice are named aging-ODEGs. Otherwise, DEGs with a lower expression level in old mice than in young mice are named aging-UDEGs.

To investigate how the DR/RALL conditions affect gene expression in the aging process, we considered the variation of expression associated with normal aging to be the baseline. If the gene was an aging-ODEG and the expression in DR was lower than in normal old mice, we defined this gene as DR-ameliorated ODEG. If the gene was an aging-UDEG and the expression in DR was higher than in normal old mice, we defined this gene as a DR-ameliorated UDEG. The method for identifying RALL-ameliorated ODEGs or UDEGs was similar with DR. The overlapping genes between DR-ameliorated DEGs and RALL-ameliorated DEGs were counted. For genes with DMR loci in the gene body and promoter, the Pearson correlation coefficients were computed between the gene expression and the bin methylation by using the scipy.stats.pearsonr function (details can be seen in Supplementary File S1).

### 2.4. GO Enrichment Analysis of DR/RALL-Related Genes

The GO enrichment of genes was analyzed using GOATOOLS (v 0.6.10) [55]. The core ontology (OBO format) was downloaded from http://www.geneontology.org/page/download-ontology (accessed on 2 November 2020) and the gene annotation was downloaded from http://www.geneontology.org/page/download-go-annotations (accessed on 2 November 2). All mice genes were downloaded from http://genome.ucsc.edu/cgi-bin/hg (accessed on 20 April 2020). Tables as background genes. The GO terms did not propagate counts to parent terms during the performance of GO enrichment analysis. The multiple test correction method was the Bonferroni correction and the test-wise α for multiple testing was 0.5. We defined GO terms with *p*-values < 0.01 as significantly enriched GO terms and filtered out GO terms that were in the top three locus levels of the biological process GO tree.

## 3. Results

### 3.1. DR and RALL Can Attenuate Gene Expression Changes Associated with Aging

Variations in gene expression are associated with the decline of cognitive and nerve cell function with aging [23]. It has been discovered that DR can alleviate the expression variations with aging in a number of genes [20,56]. In this study, the gene expressions of mice at different ages and under different conditions were compared. The samples were divided into five groups (young, old, DR, RALL, and RDRL) with three samples in each group. The gene expression data are listed in Supplementary File S2. By comparing the gene expression levels of young and old mice, we identified the genes in which the expression level changed with aging. The gene expressions between the normal aging mice and DR/RALL mice were then compared to identify the genes that potentially retard aging via dietary restriction and rapamycin treatment. From a global perspective, we investigated the genes with Fragments Per Kilobase of exon per Million reads mapped (FPKM) expression levels > 1.0, and found that the gene numbers for different ranges in the five groups were very similar (Figure S1).

**Figure 1:**
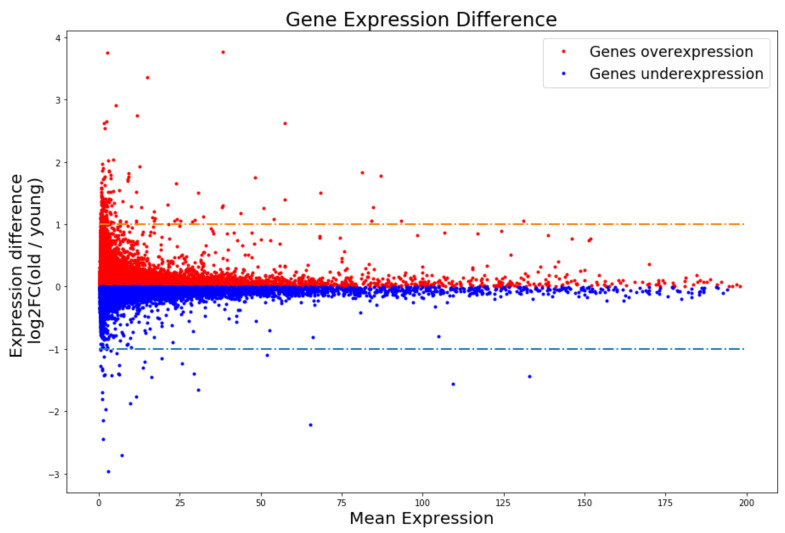
Global gene expression. Gene expression differences between young and old mice. *X*-axes means the mean expression of genes in young and old group. *Y*-axes means the log2FC of the gene expression difference between old and young group.

Genes with significantly different expression levels between two groups were defined as different expression genes (DEGs). The global expression difference between young and old mice can be seen in Figure 1. The distribution in Figure 1 indicates the fold change of most gene expression between young and old mice is smaller than 2. In addition, genes with fold changes that exceed 2 are close to the left border. That means those genes may be no expression or missing sequenced in samples. To avoid interference from these genes, we used Cufflinks (Version 2.2.1, default parameters) to compare the expression differences between young and old mice. In total, 1087 DEGs between young and old groups were identified. Compared to young mice, 839 DEGs were overexpressed in old mice (aging-ODEGs) and 248 DEGs were underexpressed in old mice (aging-UDEGs) (Table S1). Gene Ontology (GO) enrichment analysis of aging-related DEGs was executed using GOATOOLS [55]. Compared with aging-ODEGs, aging-UDEGs tended to be enriched in neurological and cognitive function GO terms, such as neuropeptide signaling pathways (GO:0007218, *p*-value = 1.72 × 10^−7^), chemical synaptic transmission (GO:0007268, *p*-value = 1.76 × 10^−7^), nervous system development (GO:0007399, *p*-value = 2.52 × 10^−7^), and axon guidance (GO:0007411, *p*-value = 8.9 × 10^−7^). Additional details can be seen in Table 1. In other words, the expression levels of genes related to neurological and cognitive function tended to diminish with aging, while aging-ODEGs tended to be enriched in non-brain specific functions. Additional details can be seen in Table 2. These results are consistent with previous literature [8,57,58,59].

**Figure 2:**
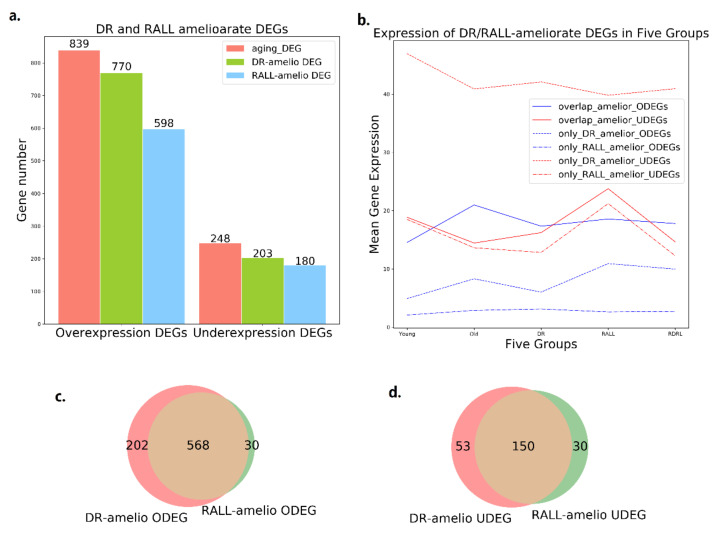
Different expression genes and DR/RALL-ameliorated genes: (**a**) number of aging-related DEGs, DR-ameliorated DEGs and RALL-ameliorated DEGs, groups distinguished by overexpression or underexpression; (**b**) mean expression of DR/RALL-ameliorated DEGs in five mice groups; (**c**) number of overlapping genes between DR-ameliorated and RALL-ameliorated ODEGs; and (**d**) number of overlapping genes between DR-ameliorated and RALL-ameliorated UDEGs.

We considered the variation of gene expression between young and old to be the baseline. In total, 770 of 839 aging-ODEGs (91.78%) remained a lower expression level in DR mice than normal old mice, named DR-ameliorated ODEGs. In addition, 203 of 248 aging-UDEGs (81.85%) remained a higher expression level in DR mice than normal old mice, named DR-ameliorated UDEGs. Overall, 89.51% (973/1087) aging-DEGs performed a better gene expression level in DR than normal aging (Figure 2a, Table S1). Through GO enrichment analysis using GOATOOLS, the DR-ameliorated UDEGs enriched in neural function-related GO terms were identified (Table S2), such as axon guidance (GO:0007411, *p*-value = 1.64 × 10^−7^), nervous system development (GO:0007399, *p*-value = 8.29 × 10^−7^), and learning (GO:0007612, *p*-value = 2.87 × 10^−6^). That is to say, dietary restriction can retard synapse- and neural-related functional decline by maintaining gene expression in a younger state. The DR-ameliorated ODEGs, however, were not directly shown to be related to neural or brain function (Table S2).

This study also identified 778 genes from 1087 aging-DEGs (71.57%) that performed a better gene expression level in old mice because of rapamycin treatment (Figure 2a, Table S1). Among the RALL-ameliorated DEGs, 180 (72.58%) underexpressed genes were ameliorated by RALL (RALL-ameliorated UDEGs), and those RALL-ameliorated UDEGs were enriched in neural function-related GO terms such as nervous system development (GO:0007399, *p*-value = 2.08 × 10^−7^), neuron migration (GO:0001764, *p*-value = 6.73 × 10^−7^), and axon guidance (GO:0007411, *p*-value = 7.99 × 10^−7^) (Table S3).

Considering that both the DR-ameliorated and RALL-ameliorated UDEGs were enriched in GO terms related to neural functions, DR and RALL may affect gene expression in a mainly overlapping gene set. We analyzed the mean expression level of overlap genes in different groups (Figure 2b). It can be seen from Figure 2b that the expression level of DR-ameliorated DEGs delay the aging-related changes because of dietary restriction. We also conducted overlap analysis for DR-ameliorated DEGs and RALL-ameliorated DEGs (Figure 2c,d, Table S4). Rapamycin treatment also has a similar effect on gene expression to protect the aging-related changes on gene expression. This study found 150 overlapping genes between the DR-ameliorated UDEGs and the RALL-ameliorated UDEGs. This indicates that DR and RALL can partially retard the aging-related expression change in the same neurally related genes (Figure 2c,d). In other words, DR and RALL can retard the neural degeneration associated with aging by maintaining the gene expression state of neurally related genes. There were 1033 DR- or RALL-ameliorated DEGs identified; 718 (69.51%) of those genes are overlapped between DR and RALL. So, rapamycin treatment could be a mimic of dietary restriction to some extent.

### 3.2. DR and RALL Can Globally Attenuate Aging-Related Methylation Changes

**Figure 3:**
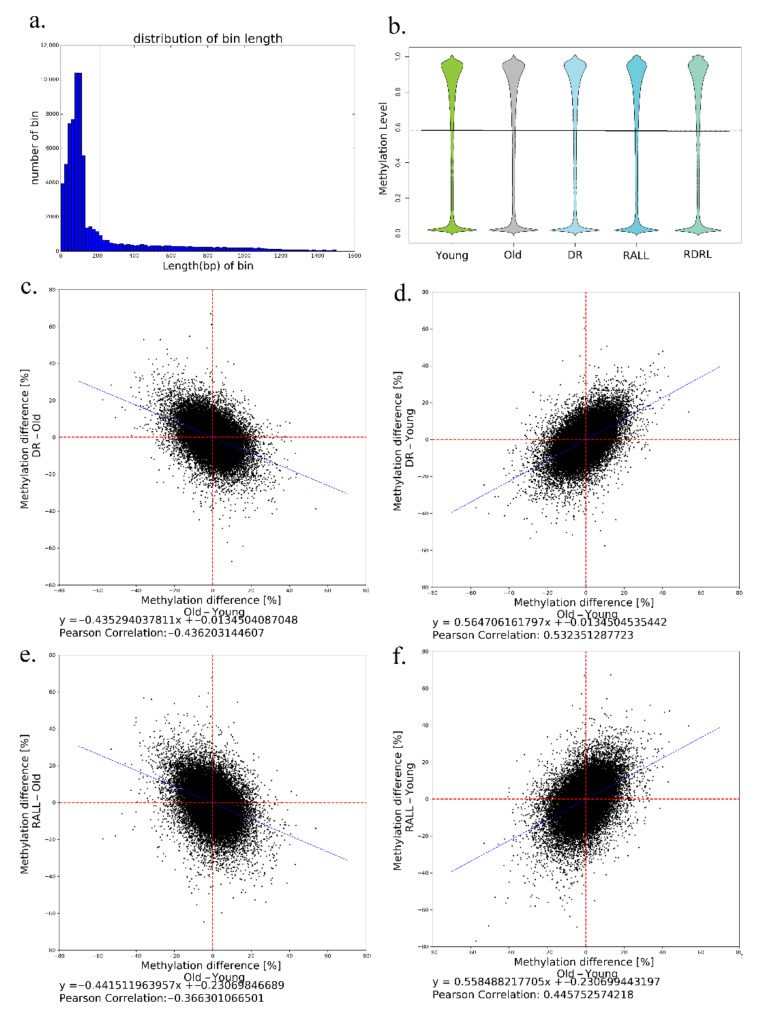
Global DNA methylation variation between different groups. (**a**) Distribution of bin length, in which the bins were created based on sequenced CpG sites, and the distance of adjacent CpG sites in one bin was no more than 1 kb; (**b**) beanplot representation of bin methylation distributions in different groups; (**c**,**e**) bin-wise methylation difference between age and (**c**) DR or (**e**) RALL; (**d**,**f**) bin-wise methylation difference between normal aging and aging controlled by DR (**d**) or RALL (**f**). The blue lines in (**c**–**f**) represent the linear regression lines. Linear regression formulas and the Pearson correlation coefficients are indicated.

This study examined the global DNA methylation level in all samples and analyzed the effects of DR and RALL conditions on DNA methylation in the aging process. The methylation levels of CpG that were sequenced using RRBS are listed in Supplementary File S3. We created bins of the genome based on sequenced CpG sites and computed the methylation levels of all bins (Supplementary File S4). The method used to divide the bins employed the procedure of Oliver Hahn et al. [2]. This study obtained a set of 72,513 bins, covering 0.82 million CpGs. Owing to the characteristics of RRBS, the bins tended to be located in CpG enrichment regions, and the average length of each bin was 212 bp (Figure 3a). We calculated a single average methylation value for all CpGs in each bin ranging from 0 (no methylation) to 1.0 (complete methylation). We analyzed the distribution of bin methylation in five groups. The methylation levels of the bins were mainly enriched by 0 (no methylation) or 0.8–1.0 (Figure 3b). The distribution of bin methylation is very similar across five groups. The distribution of bin methylation is a genome-wide statistic, but it does not mean that the methylation levels of individual bins are consistent across groups.

Despite the global DNA methylation distributions being similar in each group (Figure 3b), we wanted to determine the difference between normal aging and aging processes controlled by conditions such as DR or RALL. We considered the DNA methylation changes of normal aging to be the baseline, and the DNA methylation variations between normal aging and DR/RALL-controlled aging were computed. The coordinates of the scatterplots in Figure 3c–f indicate the differences of bin methylation between normal aging and aging controlled by DR (Figure 3c,d) or RALL (Figure 3e,f). In Figure 3c, we compared the methylation difference between normal aging and aging controlled by DR for each bin. Using linear regression, we computed the slope coefficient to be −0.4353, i.e., DR = 0.565old + 0.435young. The regression coefficients of the methylation difference indicated that the methylation level in DR mice was between the young and normal old states. In other words, from a genome-wide perspective, DR could delay changes of DNA methylation along with aging. We also compared the extent of variations between DR and young mice versus variations between young and old mice in Figure 3d, predicting that the linear regression slope coefficient was 0.565, i.e., DR = 0.565old + 0.435young, similar to the results shown in Figure 3c. We also computed the Pearson correlation coefficients for all bins, which revealed that the correlations were in accordance with the results.

For Figure 3c, the bins in the second quadrant indicate that the bins were hypomethylated in normal aging, but the methylation maintained a higher methylation level since it was controlled by DR. On the other hand, the bins in the fourth quadrant indicate that the bins were hypermethylated in normal aging, but the methylation maintained a lower methylation level since it was controlled by DR. According to the bin statistics, there were far more bins in the second and fourth quadrants than in the first and third quadrants. This observation also indicates that DR plays an active role in reducing the DNA methylation change with aging.

By analyzing the global DNA methylation levels of RALL in the same way, we also discovered that RALL can ameliorate the DNA methylation changes associated with the normal aging process. Using the linear regression of the bins, we could predict that the coefficients were −0.4415 and 0.5585, i.e., RALL = 0.5585old + 0.4415young (Figure 3e,f). The Pearson correlation coefficients were in accordance with our results. Analogous to Figure 3c,d, we could speculate that RALL could ameliorate DNA methylation based on the number of bins in each quadrant of Figure 3e,f.

By analyzing the DNA methylation levels of all bins, we found that the variability of the methylation level controlled by DR/RALL was slight compared to normal aging. In other words, from a genome-wide perspective, both dietary restriction and rapamycin treatment can ameliorate the DNA methylation changes associated with the normal aging process.

### 3.3. Aging-Related Methylation Changes Could Be Attenuated by DR and RALL

**Figure 4:**
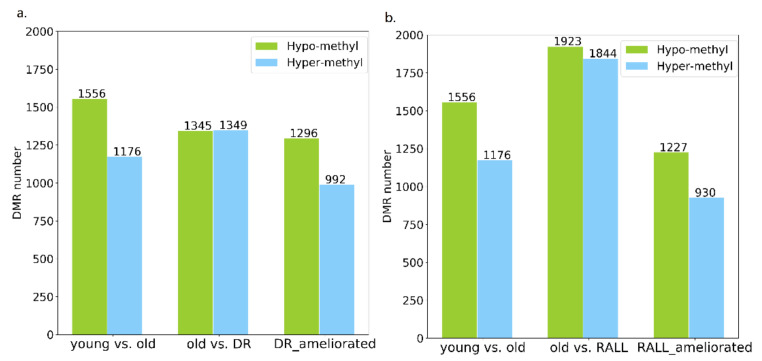
Number of DMRs and DR/RALL-ameliorated DMRs. (**a**) Number of aging-related DMRs, consisting of DR-related DMRs and DR-ameliorated DMRs; (**b**) number of RALL-related DMRs and RALL-ameliorated DMRs. The green bars represent the bins that were hypomethylated and the blue bars represent the bins that were hypermethylated.

Besides confirming that DR and RALL can globally ameliorate aging-related DNA methylation changes, we also identified in which bins the methylation changes could be ameliorated by DR and RALL during the aging process. We used the limma software package (v 3.34.9) to analyze the methylation level differences of all bins between groups of different ages. Bins with average methylation differences > 0.1 and *p*-values < 0.05 were identified as different methylation regions (DMRs) [52]. In normal aging, we identified that the methylation levels of 2732 bins exhibited significant differences between young and old mice (aging-related different methylation regions, i.e., aging-related DMRs), 1556 of which were hypomethylated and 1176 of which were hypermethylated (Figure 4, Table S5). We also analyzed whether the aging-related DNA methylation variation was influenced by dietary restriction or rapamycin treatment. Compared to normal aging mice, 2694 bins significantly changed in mice with dietary restrictions (DR-related DMRs). Among those DR-related DMRs, approximately half were hypermethylated because of dietary control, while the others were hypomethylated (Figure 4a). Compared to normal aging mice, 3767 bins significantly changed in mice treated with RALL (RALL-related DMRs) Among those RALL-related DMRs, 1844 were hypermethylated because of rapamycin control and 1923 were hypomethylated (Figure 4b).

We identified specific DNA regions and genes in which DNA methylation was significantly changed along with aging and could be ameliorated by DR. From aging-related hypermethylation bins, we selected bins in which methylation maintained a lower status in DR group, named DR-ameliorated hyper DMRs. Conversely, the aging-related hypomethylation bins, which were maintained at a higher level by DR, were defined as DR-ameliorated hypo DMRs. Both the DR-ameliorated hyper DMRs and DR-ameliorated hypo DMRs were known as DR-ameliorated DMRs; the details for identifying DR-ameliorated DMRs can be found in the Methods section. We identified 2288 DR-ameliorated DMRs, which represented 83.46% of the aging-related DMRs (Figure 4a, Table S5). For the same to DR-ameliorated DMRs, we also identified 2157 RALL-ameliorated DMRs, which represented 78.95% of the aging-related DMRs (Figure 4b, Table S5). These results indicate that DR and RALL could ameliorate methylation changes with aging in almost 80% of aging-related DMRs.

To determine which genes annotate the DR-ameliorated DMRs, we mapped those DR-ameliorated DMRs to a mice reference genome downloaded from UCSC [60]. Since the methylation of a promoter is an alternative process that will also affect gene expression [46], the genes that annotated the DR-ameliorated DMRs to both the gene body and promoter were defined as DR-ameliorated DMGs. A total of 319 DR-ameliorated hyper DMGs and 391 DR-ameliorated hypo DMGs were identified. To interpret the DR-ameliorated DMGs in the context of biological processes, we performed GO enrichment analysis of those genes using GOATOOLS [55]. The DR-ameliorated hypo DMGs and the DR-ameliorated hyper DMGs were enriched in different GO terms.

The DR-ameliorated hyper DMGs were mainly enriched in GO terms associated with nervous system function, regulation, and development, such as homophilic cell adhesion via plasma membrane adhesion molecules (GO:0007156, *p*-value = 3.09 × 10^−6^), positive regulation of synapse assembly (GO:0051965, *p*-value = 7.95 × 10^−5^), neuron migration (GO:0001764, *p*-value = 0.000307), and other processes (Table S6). DR-ameliorated hypo DMGs were enriched in GO terms such as actin cytoskeleton organization (GO:0030036, *p*-value = 5.18 × 10^−7^), intracellular signal transduction (GO:0035556, *p*-value = 1.69 × 10^−6^), and negative regulation of cell proliferation (GO:0008285, *p*-value = 5.89 × 10^−5^) (Table S6). It has been reported that altered DNA methylation levels are often associated with genes featuring tissue-specific patterns of expression [61]. Compared to DR-ameliorated hypo DMGs, DR-ameliorated hyper DMGs are inclined to be associated with GO terms having hippocampus-specific functions (Table S6).

We also identified 293 RALL-ameliorated hyper DMGs and 364 RALL-ameliorated hypo DMGs (Table S5). The RALL-ameliorated hyper DMGs were also enriched in GO terms associated with nervous system function, regulation, and development, while the RALL-ameliorated hyper DMGs were more likely to be enriched in GO terms associated with basic functions of the organism such as signal transduction, phosphorylation, animal organ morphogenesis, and immune system development (Table S7). Accordingly, DR and RALL may play a potential role in protecting brain aging by ameliorating the aging-related hypermethylation of genes.

### 3.4. DR and RALL-Ameliorated Genes Are Associated with Aging-Related Functions

**Figure 5:**
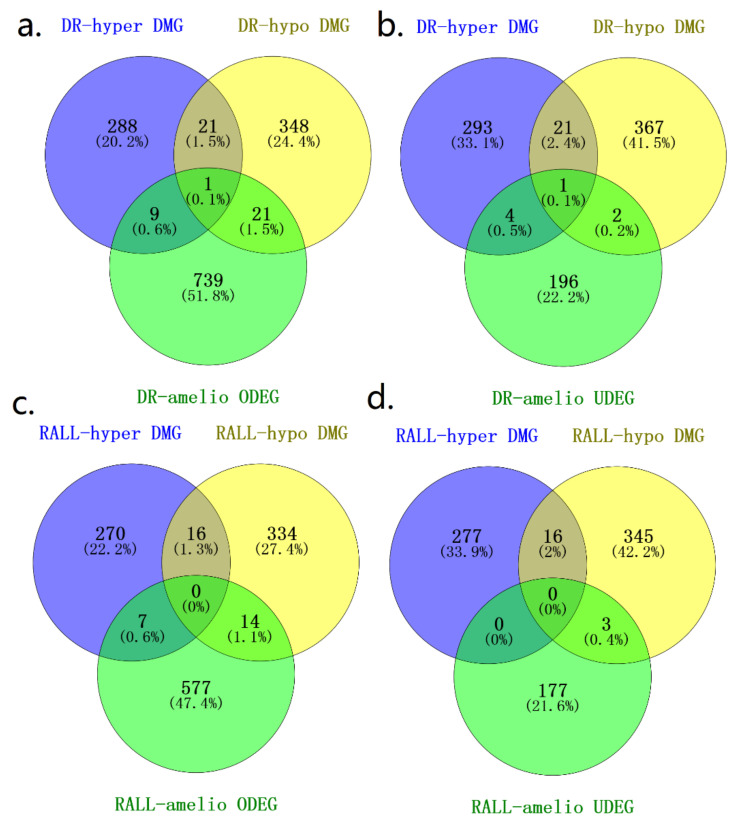
Overlap genes between DMGs and DEGs. (**a**) overlap genes between DR-ameliorated ODEGs and DR-amliorated DMGs; (**b**) overlap genes between DR-ameliorated UDEGs and DR-amliorated DMGs; (**c**) overlap genes between RALL-ameliorated ODEGs and RALL-amliorated DMGs; (**d**) overlap genes between RALL-ameliorated UDEGs and RALL-amliorated DMGs.

To investigate the potential mechanism by which DR/RALL affects aging, we not only analyzed the changed methylation state of genes but also the altered expression affected by dietary restriction or rapamycin treatment. We identified overlap genes between DR/RALL-ameliorated DMGs and DEGs (Figure 5a–d, Table S8). Furthermore, we calculated the correlation between DNA methylation and gene expression of overlap genes through all samples (details can be seen in Supplementary File S1). There are 4.2% (31/739) DR-ameliorated ODEGs altered methylation state that was due to dietary restriction (Figure 5a, Table S8 and Figure S2). Among DR-ameliorated ODEGs, only four (12.9%) genes (*Gm7120, Ifi47, Bmp7, and Ppp1r14a*) of them express significant correlation with DNA methylation (|PCC| > 0.5, *p*-value < 0.05) (Table S8 and Figure S2). We found 7/196 (3.57%) DR-ameliorated UDEGs altered methylation state that was due to dietary restriction, but the correlation between gene expression and DNA methylation of matched bin is not significant (Figure 5b, Table S8 and Figure S2). In total, 21/577 (3.64%) RALL-ameliorated ODEGs altered methylation state in RALL group (Figure 5c). Three genes (*Gm7120, Ppp1r14a, and S100b*) of RALL-ameliorated ODEGs express significant correlation with DNA methylation (Table S8 and Figure S2). We also found three RALL-ameliorated UDEGs (*Osbpl10, Clic6, Kirrel2*) overlapping with RALL-ameliorated DMGs (Figure 5d) and the expression of a significant *Osbpl10* gene correlation with methylation of chr9:115158267–115158370 (PCC = 0.714, *p*-value = 2.8 × 10^−3^) (Table S8 and Figure S2). As mentioned above, we found the overlap genes between DR/RALL-related DEGs and DMGs are very few, and the correlation between gene expression and DNA methylation is not universal. In other words, we found only a few genes in which DNA methylation and gene expression are affected by DR or RALL simultaneously.

The effect of DR and RALL on methylation is subtle. For example, one DR-ameliorated ODEG *Ms4a7* has positive correlation with chr19:11334327–11334453 (PCC = 0.232, *p*-value = 0.406), while *Ms4a7* has negative correlation with chr19:11323917–11323950 (PCC = −0.4315, *p*-value = 0.1081) (Table S8 and Figure S2). One DR-ameliorated UDEG *Cdh13* has positive correlation with DMR chr8:118821220–118821285 (PCC = 0.305, *p*-value = 0.270), while *Cdh13* has negative correlation with another DMR chr8:119221446–119221476 (PCC = −0.065, *p*-value = 0.819) (Table S8 and Figure S2). So, DR/RALL may through the affect of DNA methylation of the special region regulate gene expression, rather than by changing the methylation level of the entire gene. We also investigated genes responsible for DNA methylation in our data, for example *Dnmt1*, *Tet*, and *Mecp*. However, there is no significant change in gene expression of them among all groups (Supplementary File S2).

**Figure 6:**
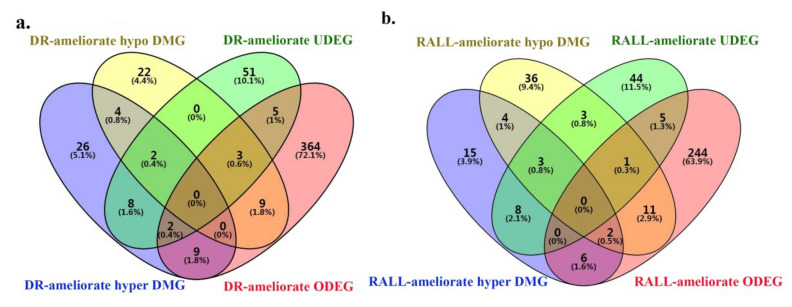
GO terms of biological processes enriched by four different gene sets of DR and RALL. (**a**) Overlapping GO terms enriched by DR-ameliorated DMGs and DR-ameliorated DEGs; (**b**) overlapping GO terms enriched by RALL-ameliorated DMGs and RALL-ameliorated DEGs. GO terms significantly enriched by genes were defined as *p*-value < 0.01. We filtered out the GO terms that were in the top three locus levels of the biological process GO tree.

Biological aging causes brain function deterioration, but DR or RALL can decrease age-related deficits in learning and memory ability [23]. To investigate which biological functions that change with aging are affected by DR/RALL in DNA methylation and can also be observed in gene expression level, we performed the GO enrichment of DR/RALL-ameliorated DEGs and DMGs. The results clearly revealed that the DR-ameliorated UDEGs and ODEGs were enriched in different GO terms (Table S2), a phenomenon that was also observed in DR-ameliorated hyper DMGs and hypo DMGs (Table S6). Interestingly, although we found that DR-ameliorated UDEGs and DR-ameliorated hyper DMGs only had a small number of overlapping genes, they both tended to be enriched in GO terms related to nervous system function. For this reason, we analyzed the GO terms that were enriched by different DR-ameliorated gene sets (Figure 6).

**Figure 7:**
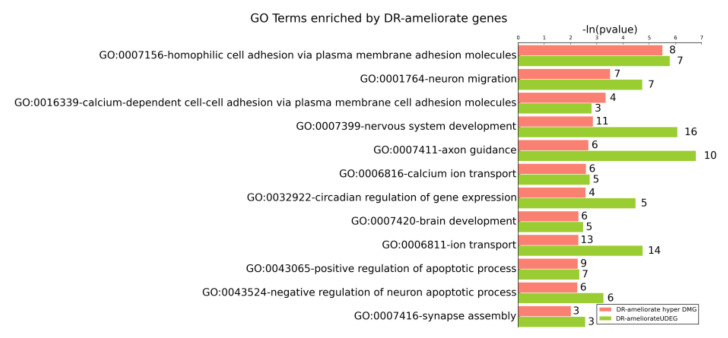
Gene ontology enriched by DR-ameliorated UDEGs and DR-ameliorated hyper DMGs. The −ln(*p*-value) and the number of genes in the GO terms are shown. The salmon-colored bars represent the GO terms enriched by DR-ameliorated hyper DMGs, while the olive-colored bars represent the GO terms enriched by DR-ameliorated UDEGs.

**Figure 8:**
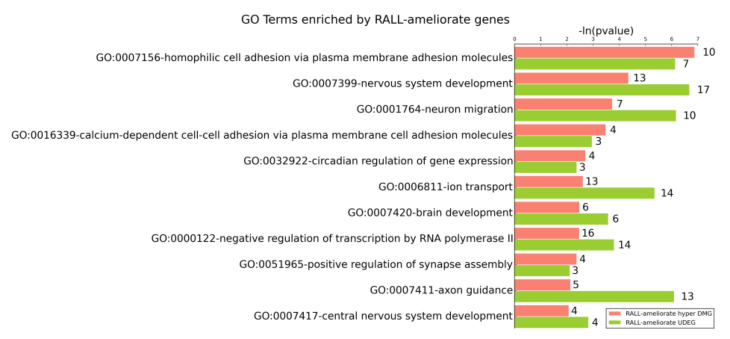
Gene ontology enriched by RALL-ameliorated UDEGs and RALL-ameliorated hyper DMGs. The −ln(*p*-value) and the number of genes in the GO terms are shown. The salmon-colored bars represent the GO terms enriched by RALL-ameliorated hyper DMGs, while the olive-colored bars represent the GO terms enriched by RALL-ameliorated UDEGs.

The DR-ameliorated hyper DMGs were significantly enriched in 51 GO terms, and it is interesting to note that 12 (23.53%) of them overlapped with GO terms in which the DR-ameliorated UDEGs were also enriched (Figure 6). This indicates that DR can affect those functions not only by ameliorating the expression of special genes, but also by ameliorating the DNA methylation of related genes. For each GO term mentioned above, however, there were almost no overlapping genes between the DR-ameliorated hyper DMGs and the DR-ameliorated UDEGs. This implies that when DR affects a function, it is not just ameliorating the DNA methylation and gene expression in the same gene, but can also affect the gene expression level and DNA methylation of different genes in the same GO terms. Those GO terms are related to functions such as brain or nervous system development, neuron function, apoptotic processes, and cell adhesion (Figure 7 and Figure 8).

**Figure 9:**
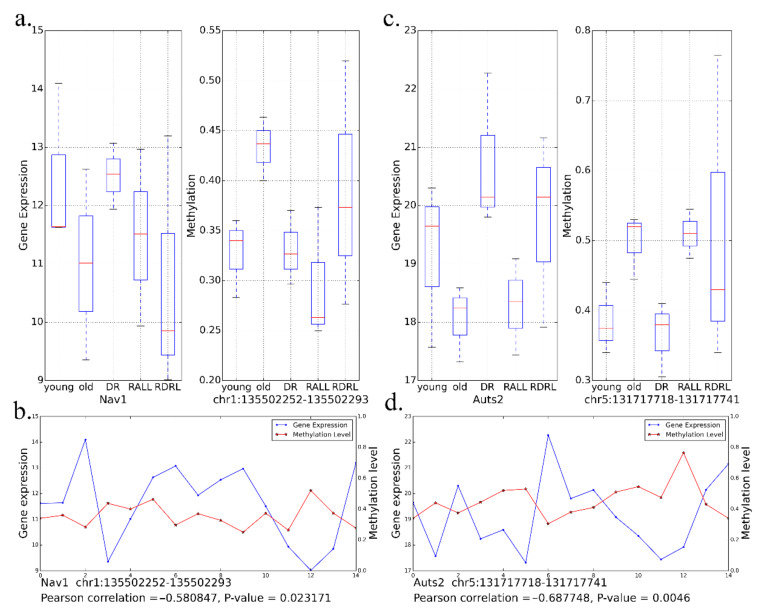
Correlation between gene expression and methylation of *Nav1* and *Auts2*. (**a**) Gene expression level of *Nav1* and methylation level of bin (chr1:135502252–135502293) in five groups; (**b**) correlation of *Nav1* expression and bin methylation in all samples; (**c**) gene expression level of *Auts2* and methylation level of bin (chr5:131717718-131717741) in five groups; (**d**) correlation of *Auts2* expression and bin methylation in all samples.

The brain development (GO:0007420) and nervous system development (GO:0007399) terms are organism development-related GO terms enriched by DR-ameliorated hyper DMGs and DR-ameliorated UDEGS. The nervous system development (GO:0007399) terms included 16 DR-ameliorated UDEGs and 11 DR-ameliorated hyper DMGs; the *p*-values of the two gene sets were 8.29 × 10^−7^ and 1.38 × 10^−3^, respectively. The brain development (GO:0007420) terms included five DR-ameliorated UDEGs and six DR-ameliorated hyper DMGs; the *p*-values of the two gene sets were 3.25 × 10^−3^ and 4.91 × 10^−3^, respectively. We computed the correlation coefficients between the gene expression and DMR methylation on genes annotated by those GO terms (Figure 9a–d, Figure S2). We computed the Pearson correlation between gene expression on each sample and methylation of DMR in matched sample by stats.pearsonr function in scipy package (v1.4.1) (details can be seen in Supplementary File S1). For example, there are 11 DR-ameliorated hyper DMGs enrichment in GO:0007399 (the nervous system development) (Table S6). Among the 11 DR-ameliorated hyper DMGs, the *Nav1* gene is the most significant gene with gene expression correlation with DNA methylation. The *Nav1* gene included a DR-ameliorated hyper DMR (chr1:135502252–135502293). The gene expression of *Nav1* was significantly correlated with the methylation of this bin (PCC = −0.5808, *p*-value = 0.0231) (Figure 9a,b, Table S8). The *Nav1* gene may play a role in the development of the human circadian system [62] and *Nav1* positively regulates neurite outgrowth and interactions [63].

In addition to the development of the brain and nervous system, there were also some GO terms related to nervous system function that were enriched by DR-ameliorated genes, such as neuron migration (GO:0001764), axon guidance (GO:0007411), and synapse assembly (GO:0007416), as seen in Figure 7. Take neuron migration (GO:0001764) for example, there are seven DR-ameliorate hyper DMGs are enriched in GO:0001764. We found the *Auts2* is the most significant gene which gene expression correlation with DNA methylation (Table S8). The expression of *Auts2* was significantly correlated (PCC = −0.6878, *p*-value = 0.0046) with the methylation of bin (chr5:131717718–131717741) (Figure 9c,d).

Cell adhesion-related GO terms such as homophilic cell adhesion via plasma membrane adhesion molecules (GO:0007156) and calcium-dependent cell-cell adhesion via plasma membrane cell adhesion molecules (GO:0016339) represent another type of function enriched by DR-ameliorated hyper DMGs and lower DEGs. Adhesion-related mechanisms involved in neuronal tissue development can be affected by the L1 family of proteins and cadherins [64]. The L1 family of proteins, including L1cam, Chl1, Nrcam, and neurofascin, are involved in neuronal migration, as well as axon growth and proper synapse formation [65]. Our data revealed that *L1cam*, *Chl1*, and *Nrcam* genes were underexpressed in old mice and DR could reduce the degree of gene expression decline to some extent (Table S1 and Supplementary File S2). Furthermore, two cadherin-related genes-*Cdh4* and *Cdh13* included one DR-ameliorated hyper DMR in each gene body. DR was also found to retard their expression changes with aging, although not significantly. We computed the Pearson correlation coefficient between the gene expression and the methylation of DMR, finding that the correlation between them was not significant. In addition, the largest mammalian subgroup of the cadherin super family of homophilic cell-adhesion proteins includes 22 genes, which were in close proximity in the chrome. We also found a DR-ameliorated hyper DMR (chr18:37835602–37835657) locus on those genes, but did not find a significant correlation between the DMR methylation and those genes.

Another category of GO terms enriched by DR-ameliorated UDEGs and DR-ameliorated hyper DMGs was involved the negative regulation of the neuron apoptotic process (GO:0043524) and the positive regulation of the apoptotic process (GO:0043065). Apoptosis, i.e., programmed cell death, plays an important role in the maintenance of cellular homeostasis [66]. GO:0043524 included six DR-ameliorated UDEGs and six DR-ameliorated hyper DMGs; the *p*-values of the two gene sets were 0.000549 and 0.00537, respectively. GO:0043065 included seven DR-ameliorated UDEGs and nine DR-ameliorated hyper DMGs; the *p*-values of the two gene sets were 0.00454 and 0.0052, respectively. In our data, however, there were no overlapping genes between the DR-ameliorated DEGs and the DMGs in the negative regulation of the neuron apoptotic process. This indicates that even though the DNA methylation affected by DR is not directly affected by the expression level of matched genes, the DR-ameliorated DMGs can also regulate the aging process along with other DR-ameliorated DEGs.

In research on aging, rapamycin is regarded as the mimic of dietary restriction since it may extend lifespan through pathways partially independent of those used by DR in invertebrate models such as Drosophila melanogaster [41] and vertebrate models such as mice [36,45,67]. We also performed GO enrichment analysis of RALL-ameliorated DMGs and DEGs. There were 11 overlapping GO terms enriched by RALL-ameliorated hyper DMGs and RALL-ameliorated lower DEGs, as seen in Figure 8. We discovered that those RALL-ameliorated functions almost coincided with the DR-ameliorated GO terms, with the exceptions of the apoptotic-related terms, calcium ion transport terms, and synapse assembly terms.

## 4. Discussion

This study examined the effects of DR/RALL on aging-related functions by analyzing DNA methylation and gene expression in mice hippocampi. Genes whose methylation or expression could be affected by DR/RALL in the aging process were identified, and the biological processes potentially affected by those genes were analyzed. The results revealed that both dietary restriction and rapamycin can retard biological aging. Both DR and RALL can slow down the aging-related changes of gene expression in some special genes. Nearly 70% of those genes are affected not only by DR but also by RALL. So DR and RALL have similar effects on aging to some extent. However, there are some genes show different variation tendency that is due to dietary restriction or rapamycin treatment.

Previous research has shown that DR and RALL can alleviate the expression changes with aging in particular genes [20,38,56,59], suggesting that DR and RALL may maintain the gene expression level in a younger state than normal aging. To examine this hypothesis, we compared the expression levels of all genes among different groups. Although most genes exhibited no significant differences between young and old, some special genes (aging-related DEGs) displayed large ranges of change in the aging process. Among the aging-related DEGs, we found that the aging-related expression variations of some genes (DR/RALL-ameliorated DEGs) were small in the DR/RALL controlled samples, and those genes were enriched in brain-related GO terms. These data suggest that DR/RALL can play a role in ameliorating the aging process. Specifically, since the expression levels of some genes, such as *Snca*, *Trem2*, and *Abca7*, were reported significantly altered in aging-related diseases [68,69,70], the DR/RALL-ameliorated DEGs in this study may play an important role in alleviating the aging process or aging-related diseases. Future studies should examine more precise gene expression trends in the normal or controlled aging process.

The DNA methylation levels of different groups were globally compared, and the variations of DNA methylation between normal aging and aging controlled by DR/RALL were examined. The DNA methylation levels exhibited no significant differences among the experimental groups in the perspective of whole genome. This finding differs from the classical observation that DNA methylation becomes hypomethylation with aging [32], although these results have been reported previously. Hadad and Unnikrishnan, for example, also found that DNA methylation remained unchanged in different-aged mice [71,72]. Using linear regression, we compared the methylation variations of all bins between normal aging and aging controlled by DR/RALL. This analysis revealed that the methylation level of DR/RALL mice maintained a value between the levels of young and old. This is similar to the results of Oliver Hahn et al. [2], whose study focused on the effect of dietary restriction on aging in the mouse liver.

Methylation variations retarded by DR and RALL have been observed in many genome regions, and those regions represent potential transcription factors [48,73]. In this study, we not only focused on the DNA methylation of single genes, but also the gene sets related to specific biological functions. First, limited by the inherent complexity of gene expression, many potential transcription factors remain unknown to researchers [74,75]. Second, the causal relationships among DNA methylation, transcription factor binding, chromatin structure, and gene expression have not been well explained [33]. Third, there are few overlap genes between DR/RALL-ameliorated DMGs and DEGs. In addition, for most of DR/RALL-ameliorated DEGs, the correlations between gene expression and methylation of matched DMR are not significant. However, it is interesting to note that the DR-ameliorated DMGs and DEGs are enriched together in some neuro-related biological functions. There were indications that DNA methylation and gene expression may collaboratively influence the aging process in an indirect manner when mice aging is controlled by either DR or RALL. Future research should examine the genes that perform biological functions in a collaborative way.

The GO enrichment analysis of DR-ameliorated DMGs and DEGs is helpful for understanding the role of DR/RALL in the aging process. Many researchers have found that DNA methylation variation occurs in tissue-specific genes [18,61]. These findings are consistent with our observation that the DR-ameliorated DMGs are related to brain or neuron functions. Nervous system development (GO:0007399) is enriched by DR-ameliorated DMGs and DEGs. This is consistent with Mattson M. P. et al., who found that DR can extend lifespan and may increase the resistance of the nervous system to age-related diseases, including neurodegenerative disorders [76]. Previous studies also found that epigenetic alternatives in the brain can affect the vulnerability of neurons and lead to afflictions such as Alzheimer’s disease (AD) [77]. In addition to increased caloric intake, low dietary folate intake can also regulate DNA methylation effects in nervous system development disorders [77]. Hence, dietary restriction may play an important role in aging by simultaneously affecting DNA methylation and gene expression.

In addition to nervous system development influenced by DR in old mice, neuron migration inactivation and synaptic deterioration are obvious phenomena in aging neurons [78,79], and synaptic damage is always associated with cognitive decline in aging and Alzheimer’s disease [80]. The *Auts2* gene is annotated by neuron migration (GO:0001764). This gene is associated with multiple neurological diseases and has been implicated as an important gene in human-specific evolution [81]. Fisher et al. found that not only sequence variations but also epigenetic changes to the *Auts2* locus could be involved in autism spectrum disorder (ASD)-related traits [82]. Therefore, the methylation of a bin in *Auts2* that is affected by DR may play a potential role in ameliorating neuron migration and brain aging.

Cell adhesion molecules are associated with several critical processes, such as cell migration and signal transduction, and are involved in memory formation within the central nervous system (CNS) [83]. Although we did not find a significant correlation between genes and methylation, the fact that DR can simultaneously ameliorate the expression and methylation of genes related to cell adhesion may indicate that DR can improve nervous system function by affecting cell adhesion. Furthermore, Rønn L. C. et al. found that cadherin-related genes decrease markedly during development [84]. In this study, we discovered that DR can ameliorate the expression changes associated with aging of some cadherin-related genes. *Cdh4* and *Cdh13* are both unconventional cadherins, which are underexpressed in the normal aging process but retard the extent of decrease by DR. Another type of protocadherin gene is always expressed in the nervous system and refers to 22 genes in close proximity in the chrome. As a result, the genes mentioned above enriched homophilic cell adhesion via plasma membrane adhesion molecules, which is a child GO term of cell adhesion. Thus, there may be an indirect relationship between gene expression and methylation.

Neuron apoptosis is a major form of neuronal cell death and is associated with aging-related neurodegeneration [85]. Neuron apoptosis in the brain differs from the apoptotic processes of other tissues, since neurons are irreplaceable. Several aging-related neurodegenerative disorders are associated with neuron apoptosis, including Alzheimer’s disease (AD), Parkinson’s disease (PD), and Huntington’s disease (HD) [77]. Many studies have reported that alterations in methylation can trigger neuron apoptosis [66] and the abnormal methylation of apoptosis-related genes can be a biomarker of cancers [66,86]. Special dietary components such as fish oil and polyphenols can protect against neuron apoptosis in aging-related afflictions such as diabetes and Alzheimer’s disease [87,88]. In other words, diet can affect the aging process by altering the neuron apoptosis process. Hence, the fact that both DR-ameliorated UDEGs and hyper DMGs are enriched in the negative regulation of neuron apoptosis indicates that DR can inhibit apoptosis by maintaining either the expression level or DNA methylation of special genes.

## 5. Conclusions

To examine the effects of DR/RALL on aging, we measured DNA methylation and gene expression in mice of different ages, either normal aging or controlled by DR/RALL. This study focused on exploring the variation of DNA methylation and gene expression related to brain aging and clarifying the role of DR/RALL in the aging process. From a genome-wide perspective, we identified the mitigation of aging by DR/RALL. In addition, special genome regions in which aging-related methylation variations could be ameliorated by DR/RALL were selected and annotated by genes. There were also some genes whose aging-related expression variation could be ameliorated by DR/RALL. Through analysis of the overlap of DR/RALL-ameliorated DEGs, we can infer that DR and RALL have similar effects on aging to some extent. Using GO enrichment analysis of those genes, we discovered that DR/RALL can affect aging-related functions of the brain, such as brain development, the neuron apoptotic process, neuron function, and cell adhesion. Meanwhile, we found genes such as *Nav1* and *Auts2* whose expression could potentially be regulated by DNA methylation. Through the correlation of DR/RALL-ameliorated DMGs and DEGs and GO enrichment analysis, we can infer that DR and RALL ameliorate the aging process by jointly affecting both DNA methylation and gene expression, which is an important consideration for further epigenetic aging studies.

## Figures and Tables

**Table 1:** Top 10 GO terms enriched by aging-UDEGs.

GO	Name	#Gene	*p*-Value	Bonferroni
GO:0007218	neuropeptide signaling pathway	8	1.72 × 10^−7^	0.00204
GO:0007268	chemical synaptic transmission	11	1.76 × 10^−7^	0.00208
GO:0007399	nervous system development	19	2.52 × 10^−7^	0.00299
GO:0016339	calcium-dependent cell-cell adhesion via plasma membrane cell adhesion molecules	6	3.34 × 10^−7^	0.00397
GO:0007156	homophilic cell adhesion via plasma membrane adhesion molecules	10	4.69 × 10^−7^	0.00557
GO:0006811	ion transport	18	5.57 × 10^−7^	0.00662
GO:0001764	neuron migration	10	7.35 × 10^−7^	0.00873
GO:0007411	axon guidance	14	8.90 × 10^−7^	0.0106
GO:0044331	cell-cell adhesion mediated by cadherin	5	8.93 × 10^−7^	0.0106
GO:0034332	adherens junction organization	5	2.42 × 10^−6^	0.0287

#Gene: Number of aging-UDEGs enriched in GO term.

**Table 2:** Top 10 GO terms enriched by aging-ODEGs.

GO	Name	#Gene	*p*-Value	Bonferroni
GO:0042590	antigen processing and presentation of exogenous peptide antigen	5	6.42 × 10^−8^	0.000762
GO:0042270	protection from natural killer cell mediated cytotoxicity	5	6.42 × 10^−8^	0.000762
GO:0042832	defense response to protozoan	11	7.56 × 10^−8^	0.000897
GO:0007159	leukocyte cell-cell adhesion	10	9.21 × 10^−8^	0.00109
GO:2000406	positive regulation of T cell migration	7	1.10 × 10^−7^	0.00131
GO:0001516	prostaglandin biosynthetic process	8	1.10 × 10^−7^	0.00131
GO:0016064	immunoglobulin mediated immune response	7	1.10 × 10^−7^	0.00131
GO:0070098	chemokine-mediated signaling pathway	12	1.31 × 10^−7^	0.00155
GO:0034341	response to interferon-γ	10	1.32 × 10^−7^	0.00157
GO:0032088	negative regulation of NF-kappaB transcription factor activity	12	1.72 × 10^−7^	0.00204

#Gene: Number of aging-ODEGs enriched in GO term.

## Supplementary Materials

The following supporting information can be downloaded at https://www.mdpi.com/article/10.3390/genes13040699/s1: Supplementary File S1: Processing of DNA methylation and Gene expression; Table S1: Different Expression Genes and DR_RALL ameliorate Genes.xlsx; Supplementary File S2: Gene expression.txt; Figure S1: Figure of distribution of genes in expression range.pdf; Table S2: GO Enrichment of DR-DEGs.xlsx; Table S3: GO Enrichment of RALL-DEGs.xlsx; Table S4: Expression of DR-RALL-ameliorated-DEGs.xlsx; Supplementary File S3: CpG_methylation_level.txt; Supplementary File S4: Bin_locus_info.txt; Table S5: Different Methylation Region and Genes.xlsx; Table S6: GO Enrichment of DR-related DMG.xlsx; Table S7: GO Enrichment of RALL-related DMG.xlsx; Table S8: Correlation of methylation and gene expression of overlap DEGs and DMGs.xlsx; Figure S2: Figures of Gene Expression and DMR methylation.pdf.
