# Supplementary material for: Dietary Restriction and Rapamycin Affect Brain Aging in Mice by Attenuating Age-Related DNA Methylation Changes
Source: Genes (Basel). 2022 Apr 15;13(4):699. doi: 10.3390/genes13040699 (PMC9030181; doi:10.3390/genes13040699)
Supplement: Supplementary file 1 [file genes-13-00699-s001.zip › Figure S1 - Figure of distribution of genes in expression range.pdf]

Supplement file S2: Dstribution of genes in expression range

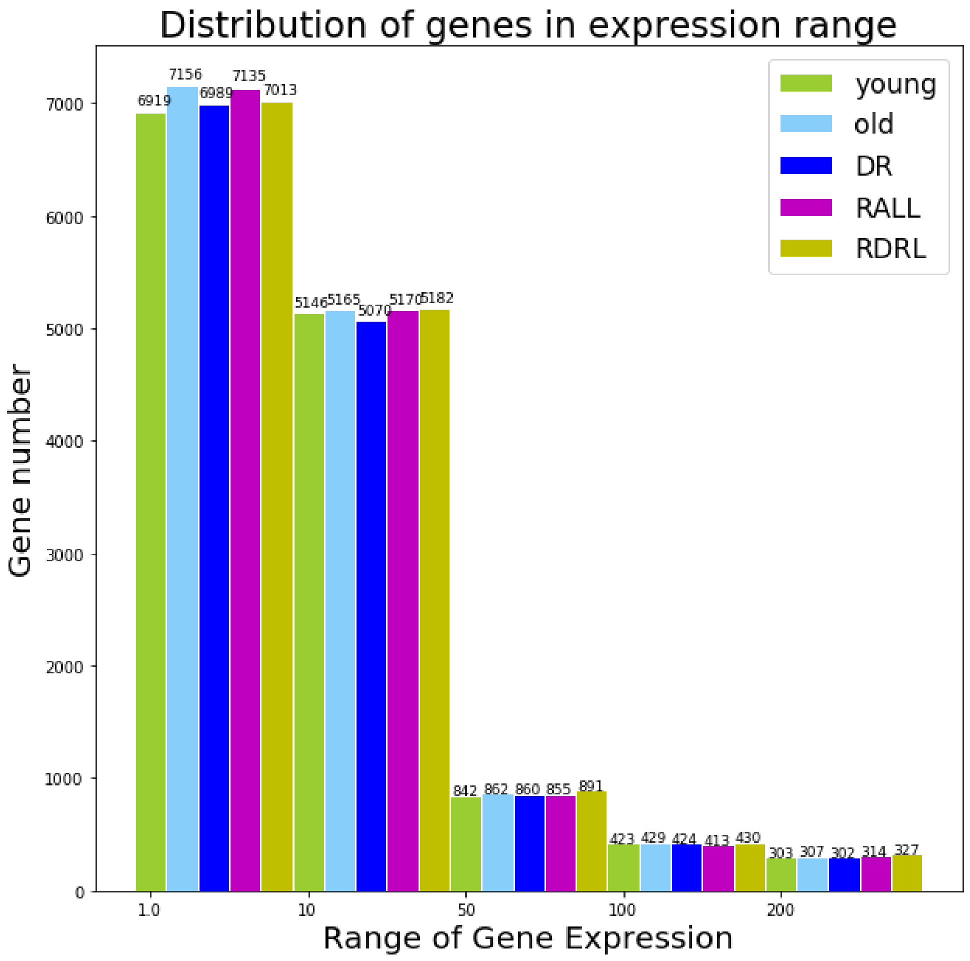

Figure s1 Distribution of genes in expression range. Number of genes in different ranges of expression for each group.
