# Supplementary material for: Dietary Restriction and Rapamycin Affect Brain Aging in Mice by Attenuating Age-Related DNA Methylation Changes
Source: Genes (Basel). 2022 Apr 15;13(4):699. doi: 10.3390/genes13040699 (PMC9030181; doi:10.3390/genes13040699)
Supplement: Supplementary file 1 [file genes-13-00699-s001.zip › Supplementary File S1 - Processing of DNA methylation and Gene expression.pdf]

Supplement S1 - Processing of DNA methylation and Gene expression

DNA methylation data analysis after Sequencing

The *.fastq files were aligned to the mouse genome (GRCm38) using BS-Seeker2 (v 2.1.1, max-mismatch = 4) [1] and bowtie2 (v 2.0.0, default parameters) [2]. The methylation levels of the CpG sites were calculated from the *.bam files generated by BS-Seeker2. The read number and methylation level of each CG site were included in the result files. We filtered out the non-CpG sites and CpG sites with read numbers <5. We then divided all sequenced CpG sites into scattered bins. The maximum distance between two adjacent sites was no more than 1 kbp in each bin. The methylation level of each bin was the average methylation level of all sites in that bin. We computed the methylation difference of bins between two groups using limma package (v 3.34.9) [3]. Bins with p-values <0.05 and average methylation differences >0.1 were identified as significant different methyla-tion regions (DMRs).

Step 1: Prepare the reference genome.
The reference mice genome (GRCm38) downloaded from UCSC.
https://hgdownload.soe.ucsc.edu/downloads.html#mouse

Step 2: Build the index for BS-Seeker2.
Pre-processing the reference genome is required only once, and the running time highly depends on the size of the genome. The command to index RRBS reads using BS-Seeker2 is:
python /BS-Seeker2/BSseeker2-master/bs_seeker2-build.py
-f /mouse_reference_genome_mm10/mm10_genome.fa --aligner=bowtie

Step 3: Map reads on 3-letter converted genome.
Take sample_1 as an example. Prepare *sample_1.fastq* first. Then set the reference genome to *genome.fa*, aligner to *bowtie2*, 4 mismatches in one read and the output file to *sample_1.bam*. The command to e
python /BS-Seeker2/BSseeker2-master/bs_seeker2-align.py
-i sample_1.fastq --aligner=bowtie2 -o sample_1.bam -f bam –m 4
-g /mouse_reference_genome_mm10/genome.fa

Step 4: This step calls methylation levels from the mapping result.
Set the *sample_1.bam* file generated in the previous step to the input file, and the output file to *sample_1*. Finally, the *.ATCGmap file is generated.
python /BS-Seeker2/BSseeker2-master/bs_seeker2-call_methylation.py
-i sample_1.bam -o sample_1
-d /BSseeker2-2.0.0/bs_utils/reference_genomes/mm10_genome.fa

Step 5: We filtered out the non-CpG sites and low quality CpG sites.

*.ATCGmap file of each sample are obtained from step 4. We filtered out the non-CpG sites and CpG sites with mapping read numbers < 5. Then we collect the CpG sites overlaped in all samples. The processing flow of Step 5 is shown in Figures 1 and 2.

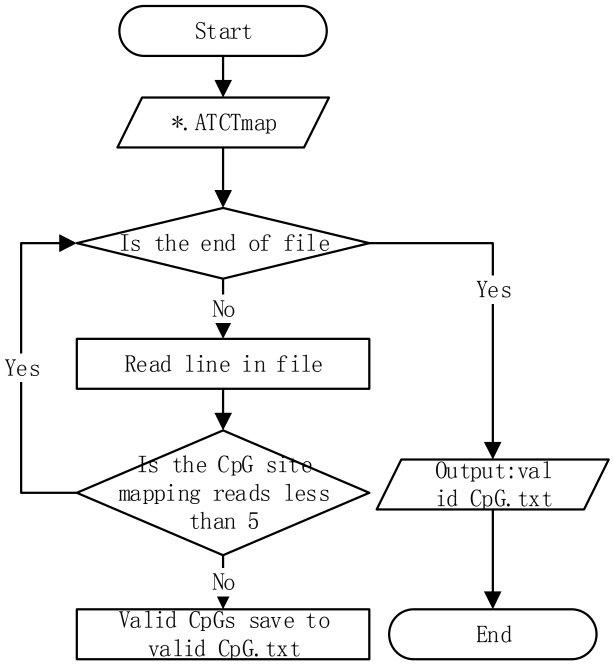

Figure 1 Valid CpG sites are obtained by filtering

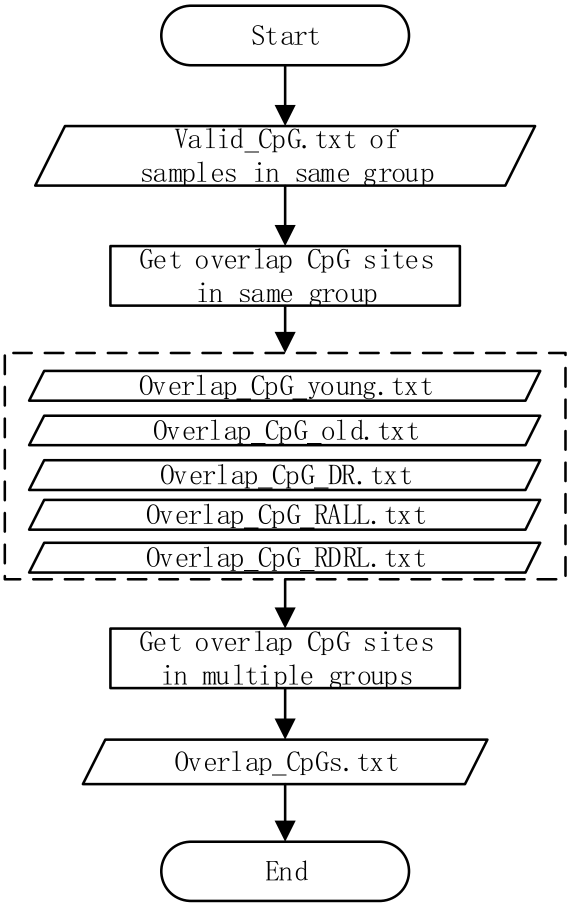

Figure 2 Overlap CpG sites obtained from multiple samples

Step 6: All sequenced CpG sites are divided into scattered bins.
We successively scanned all CPG sites, if the distance between current site and the previous CpG site was no more than 1 kbp, we put current CpG site into current bin. Otherwise, set the site to the start of a new bin. The locus of bins can be seen in Supplement S9 - Bin_locus_info.txt. We calculated mean methylation value for all CpGs in each bin ranging from 0 (no methylation) to 1.0 (complete methylation). The processing flow of Step 5 is shown in Figures 3.

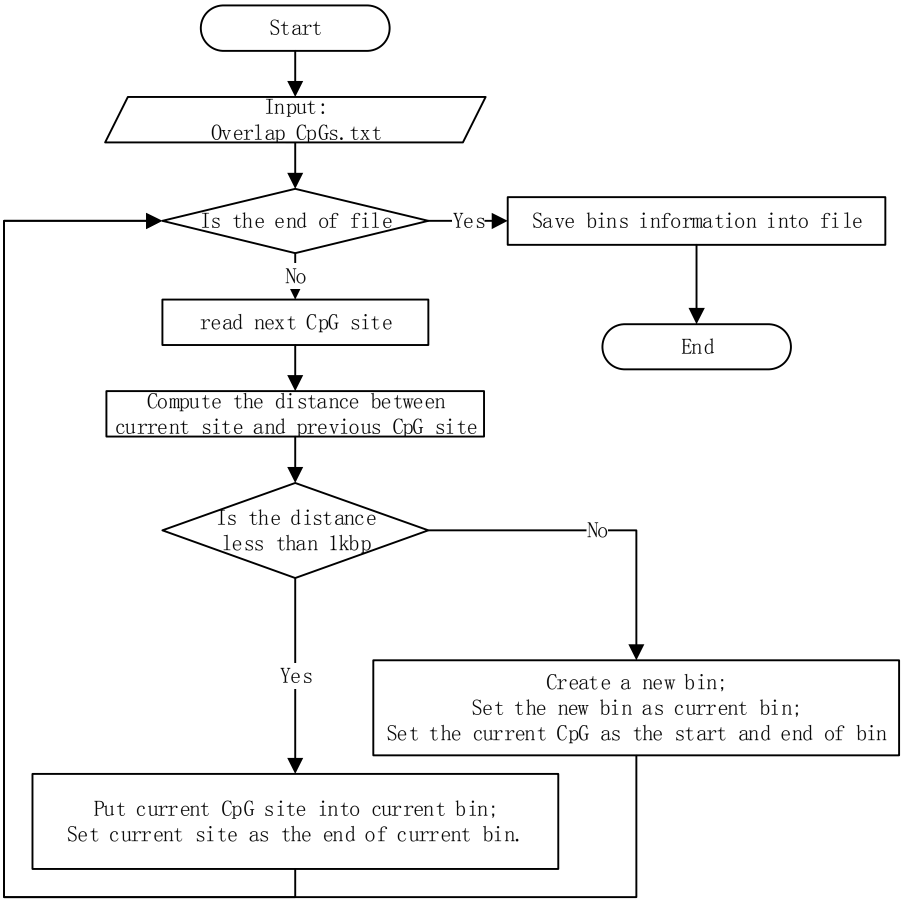

Figure 3 The process of creating scattered bins

Step 7: DMRs are identified by using limma package.
We computed the methylation difference of bins between two groups using limma package (v 3.34.9) of R. Bins with p-values <0.05 and average methylation differences >0.1 were identified as significant different methyla-tion regions (DMRs). Take the aging-related DMR identified from compare between young and old for example, the key scripts are as follows:

```
library("limma")
for (chr_index in 1:22)
{
   if (chr_index == 20)
   chr_index <- 'M'
   if (chr_index == 21)
      chr_index <- 'X'
   if (chr_index == 22)
```

```
    chr_index <- 'Y'
  y <- read.table(paste("chr",as.character(chr_index),"_bin_methy_list.txt",sep = "",collapse=""),header = TRUE,sep = "")
  bin_id <-y[,1]
  y1<-as.matrix(y[,c(2,3,4,5,6,7)]) #Young vs. Old
  filename1 = "Young_Old_chr"
  rownames(y1) <- paste(bin_id)
  design <- cbind(Grp1=1,Grp2vs1=c(0,0,0,1,1,1))
  fit <- lmFit(y1,design)
  fit <- eBayes(fit)
  y1_dim <- dim(y1)
  res <- topTable(fit, number = y1_dim[1],coef=2)
  write.table(res, file = paste(filename1,as.character(chr_index),"_limma_lmfit_res.txt",sep = "",collapse=""), row.names
= T, quote = F, sep="\t")
}
```

Gene expression data processing after Sequencing

The *.fastq files were aligned using Tophat (v 2.1.1, default parameter) [56] in order to generate the *.bam file and the reference genome (GRCm38) downloaded from UCSC. The *.bam files were then processed using Cufflinks (v 2.2.1) in order to obtain the expression level of each gene. The FPKM values of the genes were used in further analysis. The different expression genes between the two groups were identified using Cuffdiff and the genes with p-values <0.05 were selected as different expression genes (DEGs). DEGs list and the FDR-adjusted p-value of the test statistic can be seen in supplement S3.

Step 1: Make and install Topat-2.1.1.tar.gz.
./configure --prefix=/home/tophat_dir/ --with-boost=/home/tophat_boost_dir/
make
make install

Step 2: Prepare the reference genome.
The reference mice genome (GRCm38) downloaded from UCSC.
https://hgdownload.soe.ucsc.edu/downloads.html#mouse

step3: Build index of mice by using bowtie2.
/home/bowtie2/bowtie2-build mm10_genome.fa mouse_index

Step 4: Running Tophat to obtain *.bam files.
tophat /home/mouse_reference_genome_mm10/mouse_index sample_1.fastq

Step 5: Obtain the gene expression of genes.
/home/cufflinks-2.2.1.Linux_x86_64/cufflinks
-g /home/mice_refer_genome/mm10_genome.gtf
-o /home/cufflinks-2.2.1.Linux_x86_64/sample_1_cuff_out
/home/mrna-seq/tophat_out_sample_1/sample_1_accepted_hits.bam

Step 6: The expression differences among multiple samples were compared by using cuffdiff.
./cuffdiff -o /home/mrna-seq/cuffdiff_out
-L Young,Old

/home/cufflinks-2.2.1.Linux_x86_64/merge_all_out/merged.gtf
/home/cufflinksdata/sample_1_accepted_hits.bam,
/home/cufflinksdata/sample_2_accepted_hits.bam,
/home/cufflinksdata/sample_3_accepted_hits .bam
/home/cufflinksdata/sample_4_accepted_hits.bam,
/home/cufflinksdata/sample_5_accepted_hits.bam,
/home/cufflinksdata/sample_6_accepted_hits.bam

Pearson Correlation Coefficients between the gene expression and methylation

We have got locus of all bins, as shown in Figure 3 and Supplement S9 - Bin_locus_info.txt. We obtained the locus of all genes using UCSC_genes as reference gene (download from UCSC |Tools|Table Browser, http://genome-asia.ucsc.edu/index.html). Bins located in the gene body and promoter are then identified according to the location information of genes and bins. We took the gene expression level of the gene in all samples as list 1. We took the methylation level of bin (located within gene body and promoter) in all samples (in the same order as the gene expression samples) as list 2. We computed the pearson correlation between list 1 and list 2 by stats.pearsonr function in scipy package (v1.4.1). Correlation of methylaion and gene expression of overlap DEGs and DMGs can be seen in Supplement S13.

References

[1] Guo, W.; Fiziev, P.; Yan, W.; Cokus, S.; Sun, X.; Zhang, M. Q.; Chen, P. Y.; Pellegrini, M., BS-Seeker2: a versatile aligning pipeline for bisulfite sequencing data. *BMC genomics* **2013,** *14* (1), 774.

[2] Langmead, B.; Salzberg, S. L., Fast gapped-read alignment with Bowtie 2. *Nature methods* **2012,** *9* (4), 357-359.

[3] Ritchie, M. E.; Phipson, B.; Wu, D.; Hu, Y.; Law, C. W.; Shi, W.; Smyth, G. K., limma powers differential expression analyses for RNA-sequencing and microarray studies. *Nucleic acids research* **2015,** *43* (7), e47.
