# Supplementary figures and images for: Dietary Restriction and Rapamycin Affect Brain Aging in Mice by Attenuating Age-Related DNA Methylation Changes
Source: Genes (Basel). 2022 Apr 15;13(4):699. doi: 10.3390/genes13040699 (PMC9030181; doi:10.3390/genes13040699)

Supplement S12 Figures of Gene Expression and DMR methylation

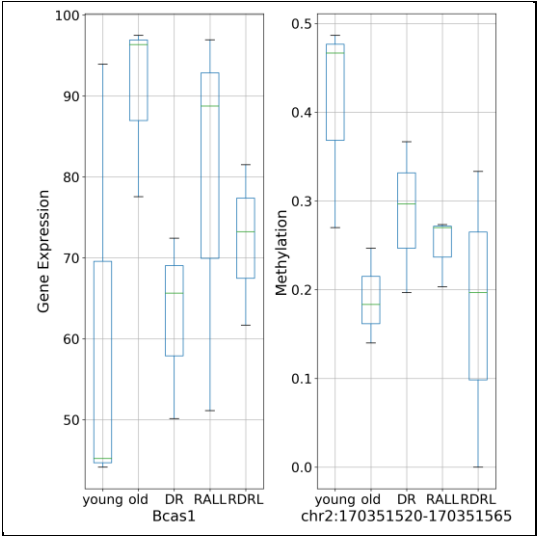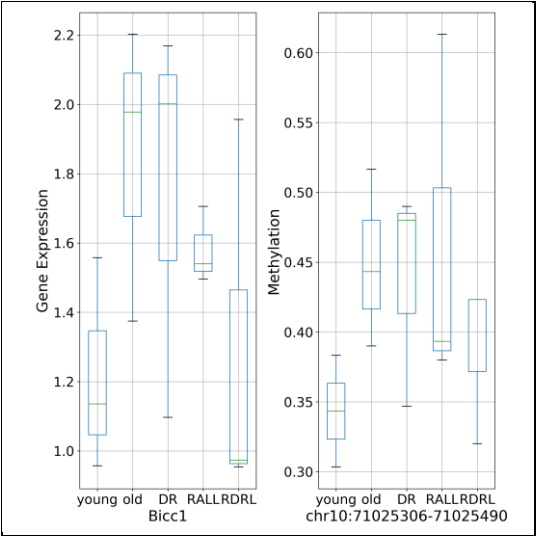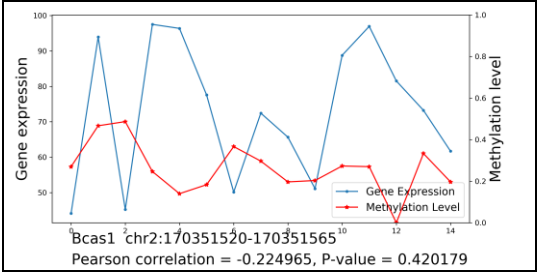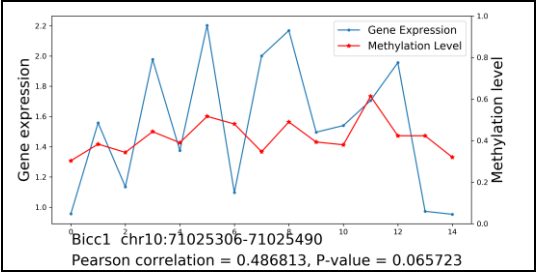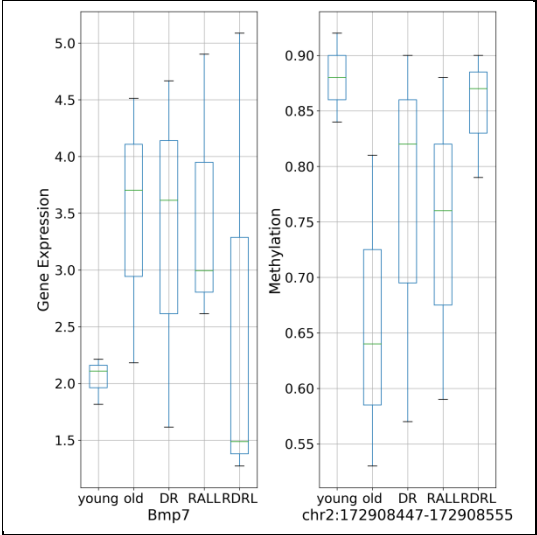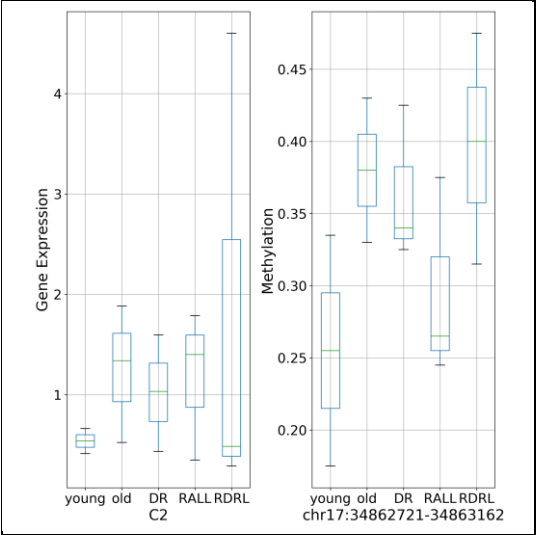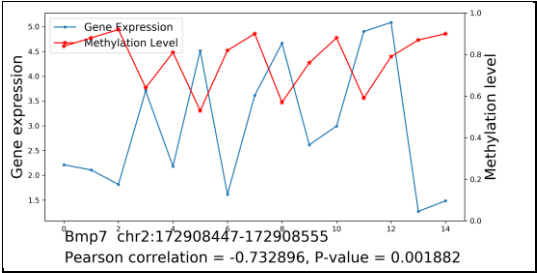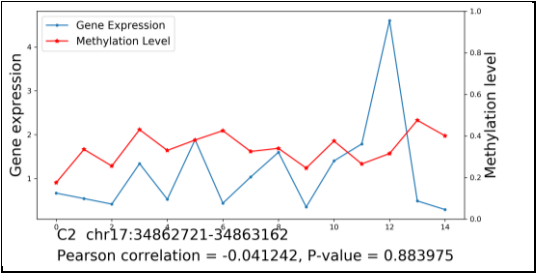

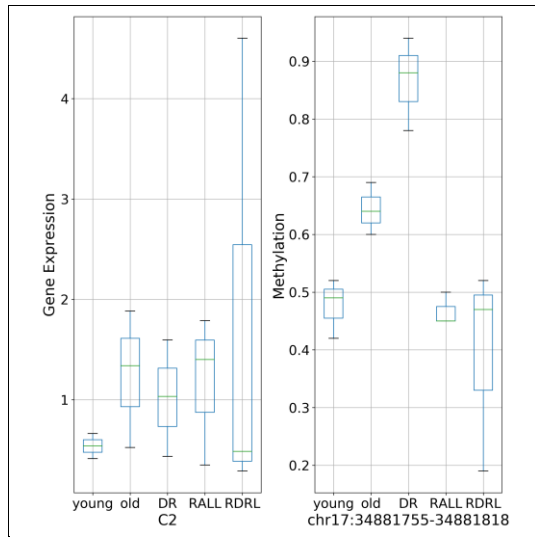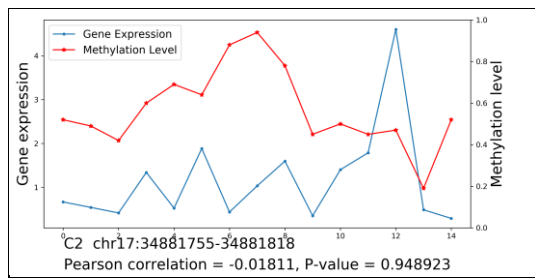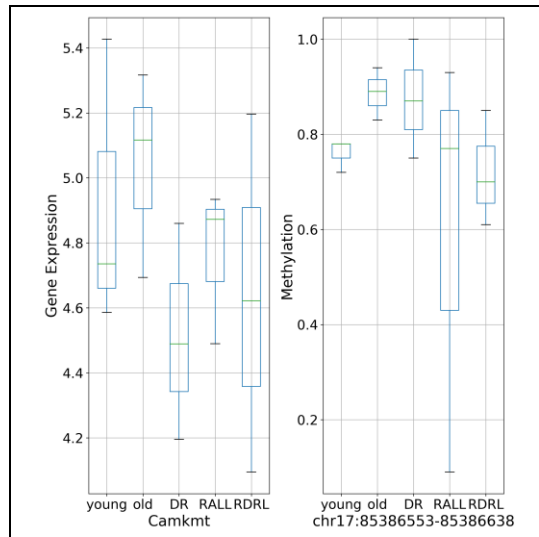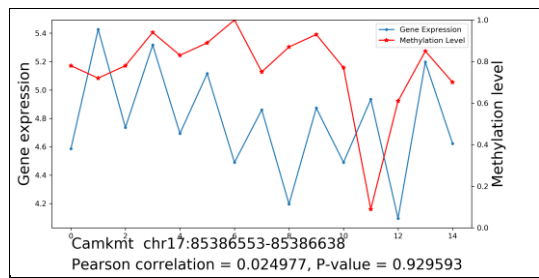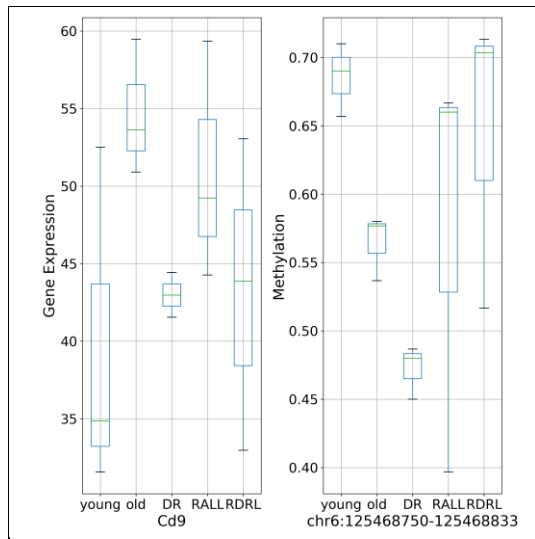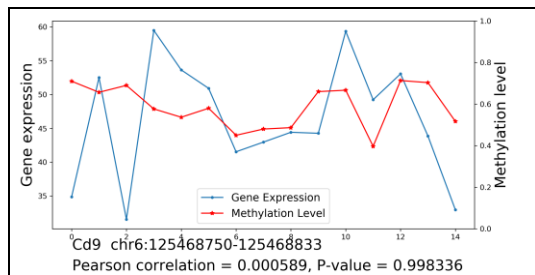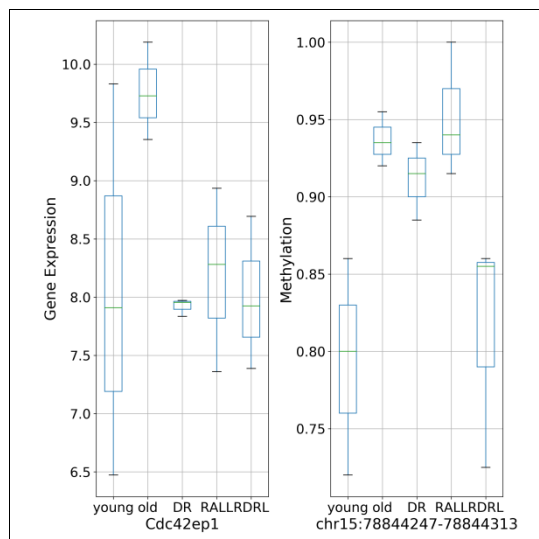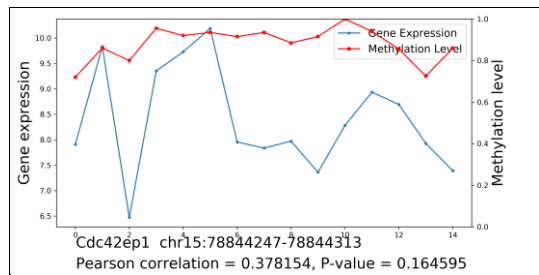

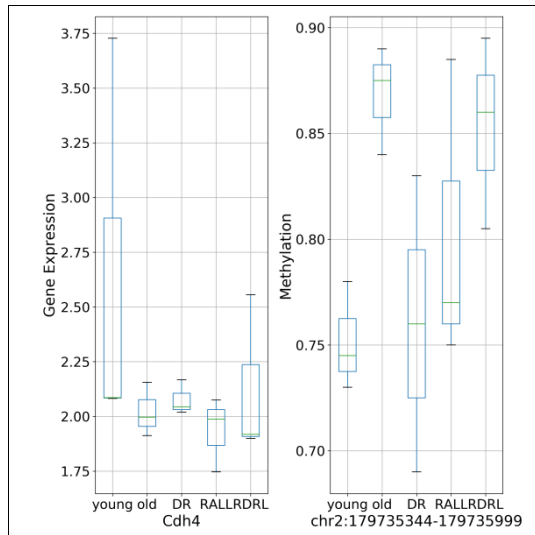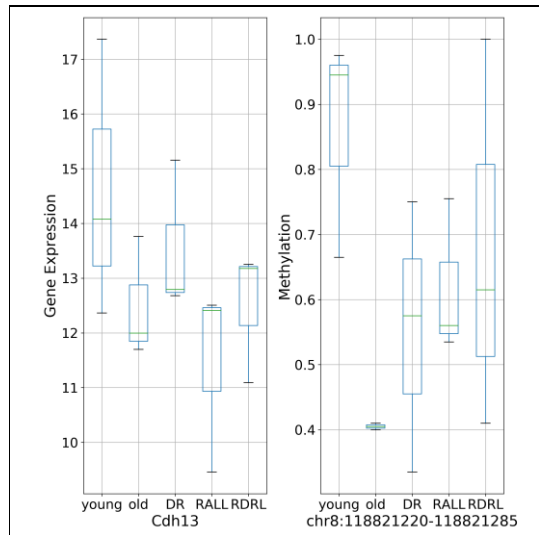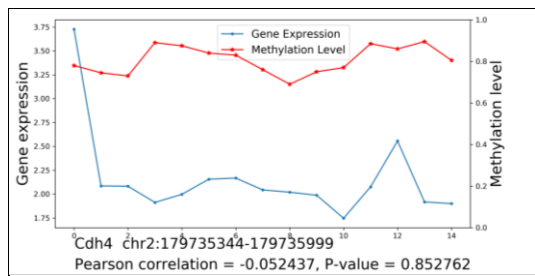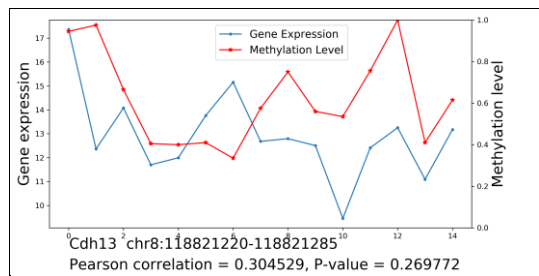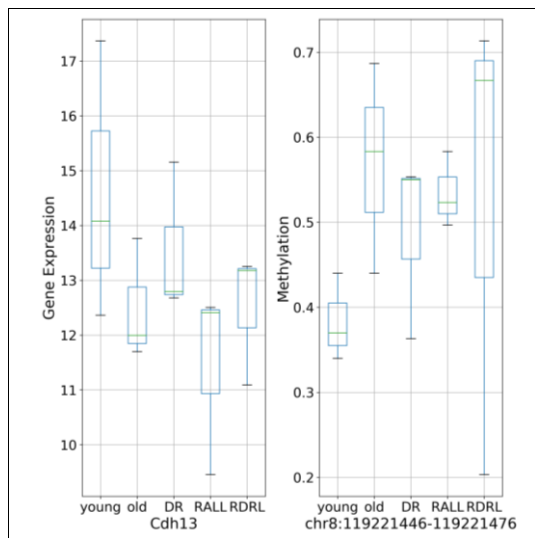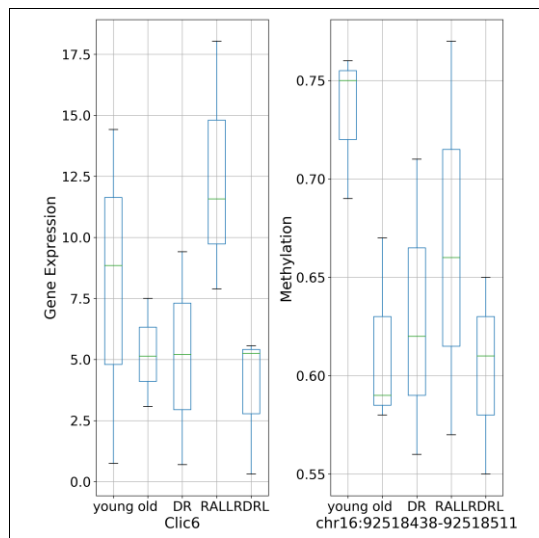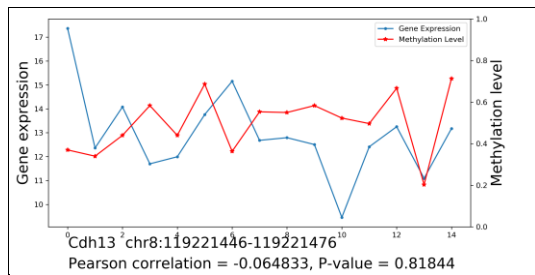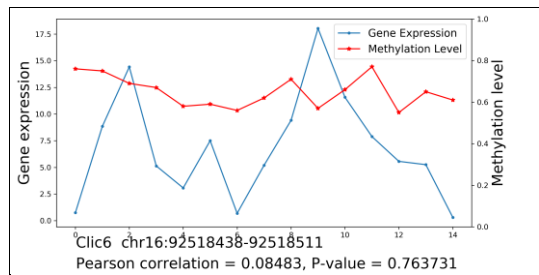

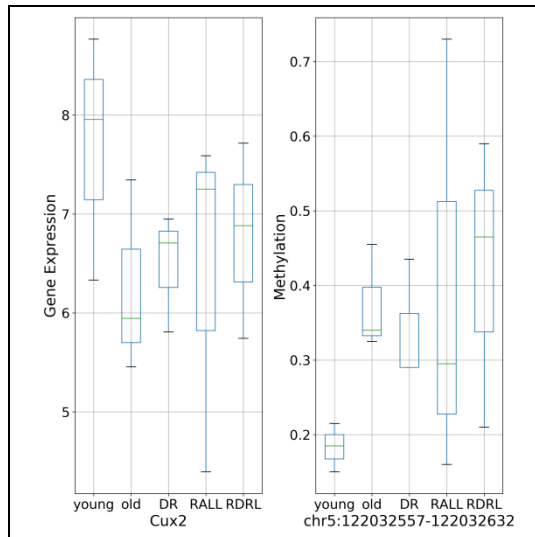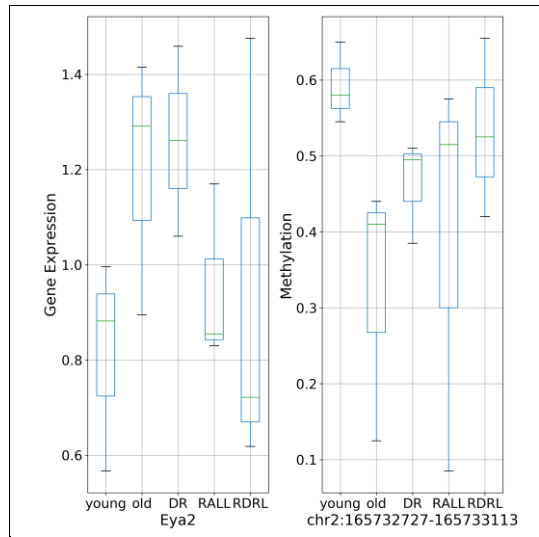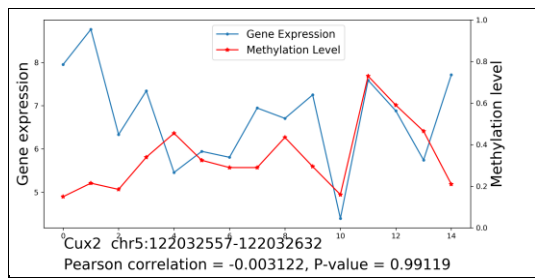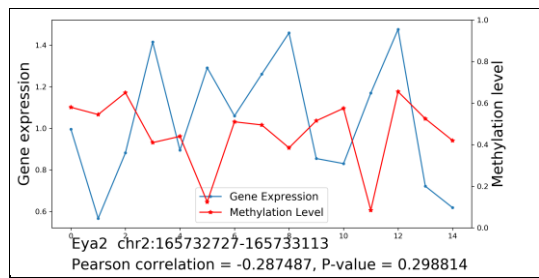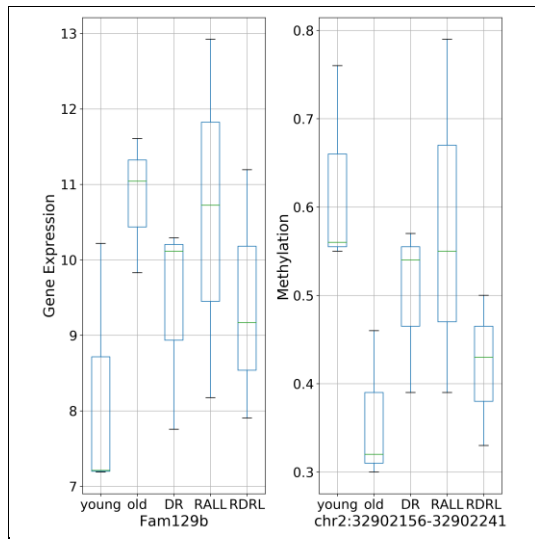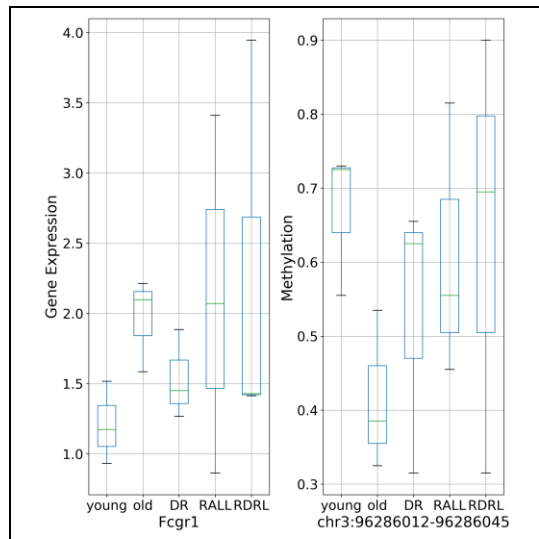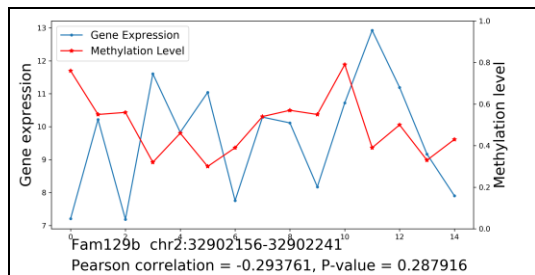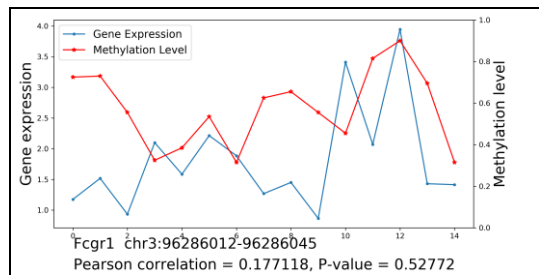

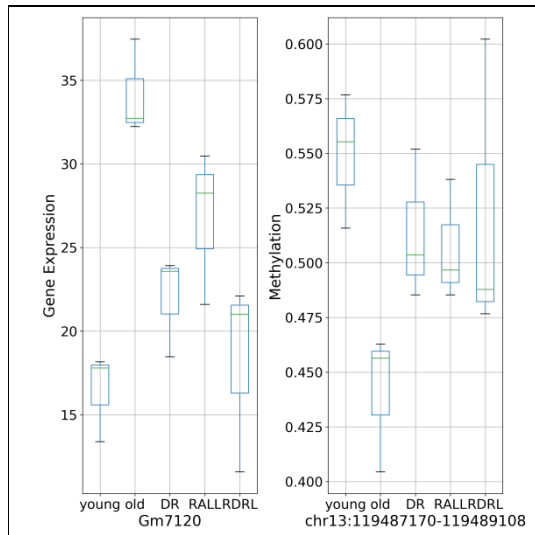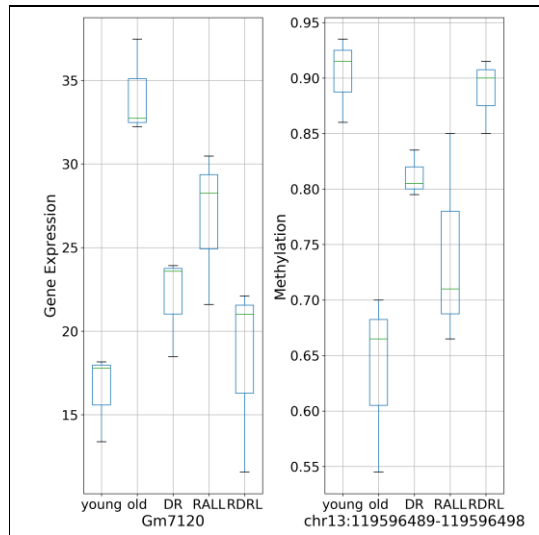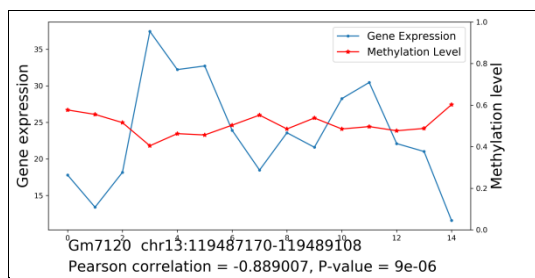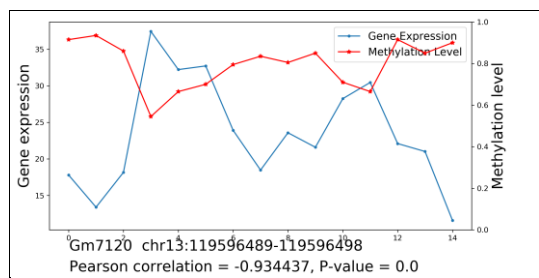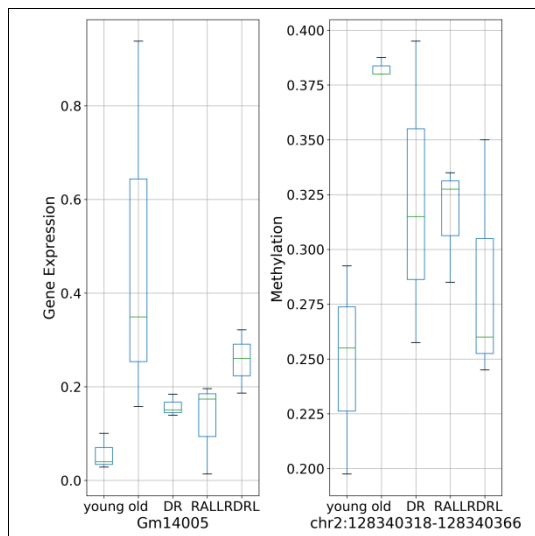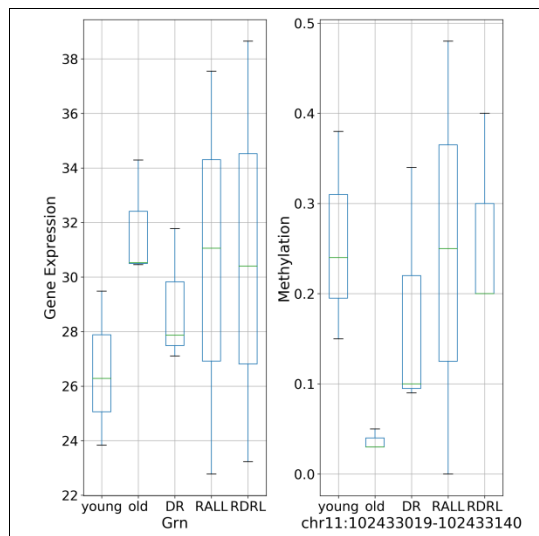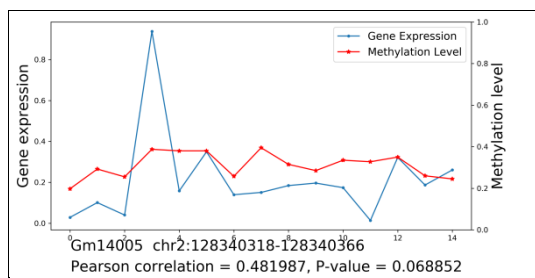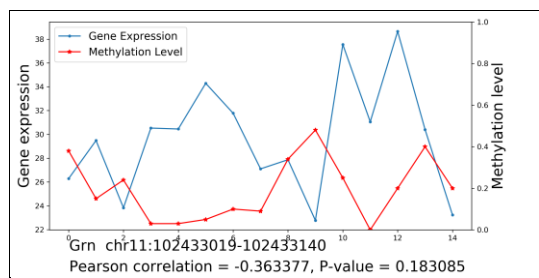

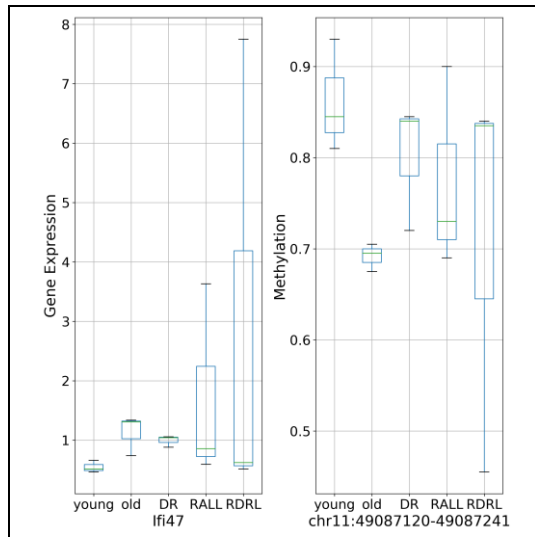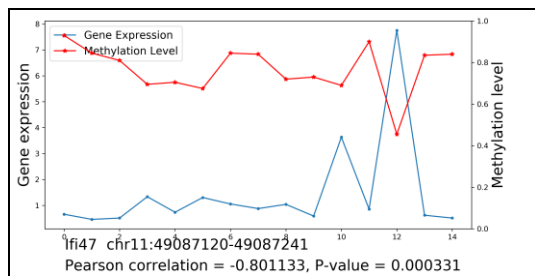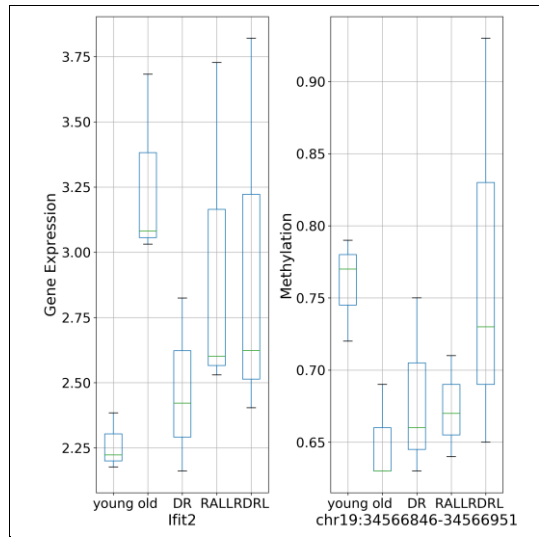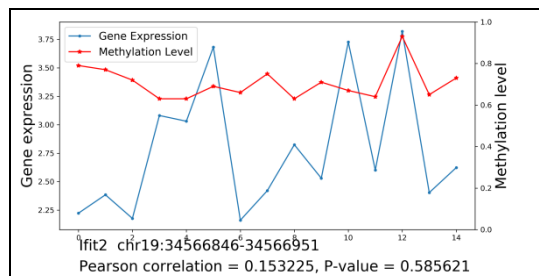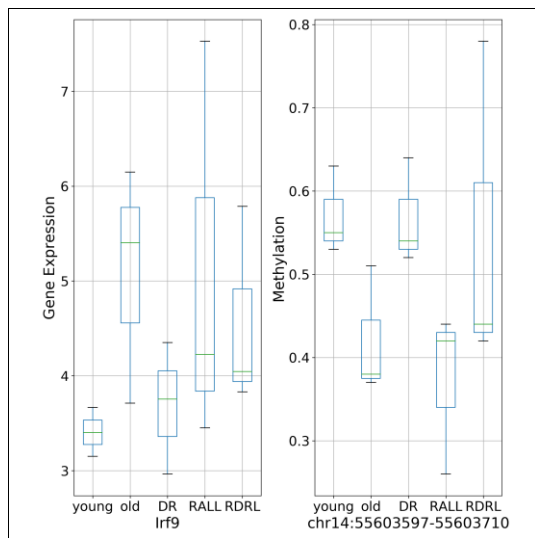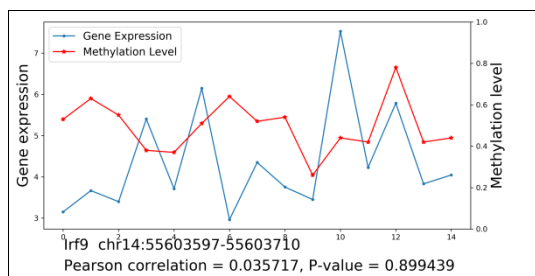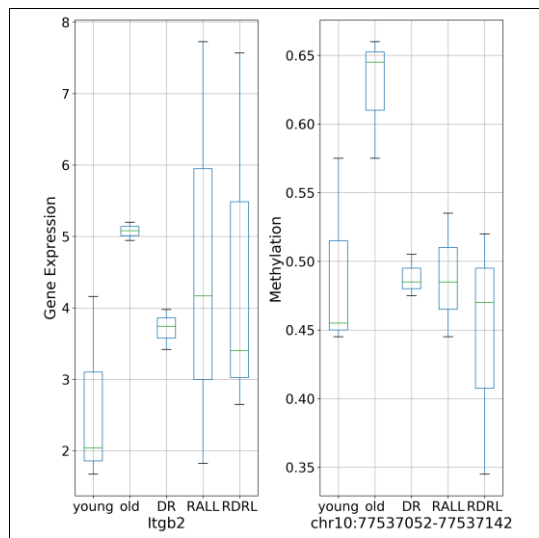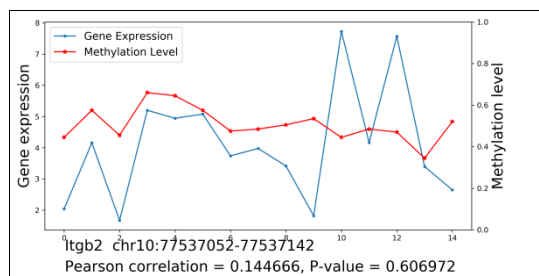

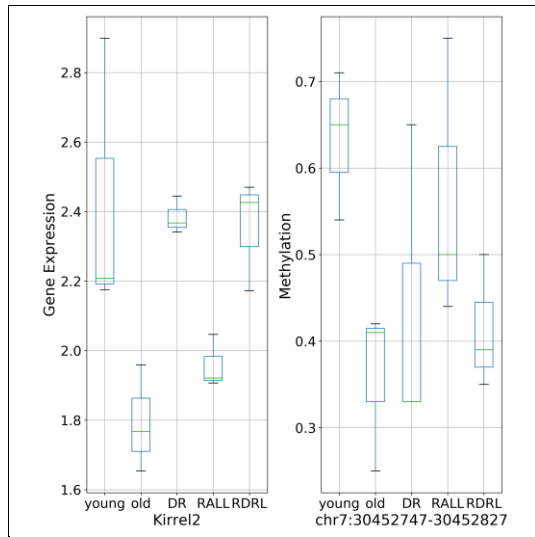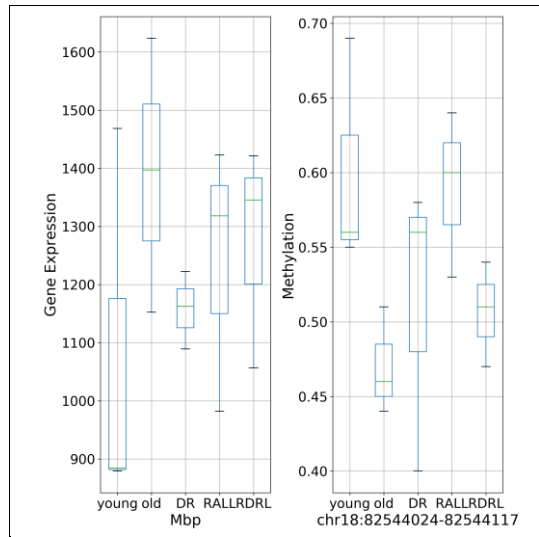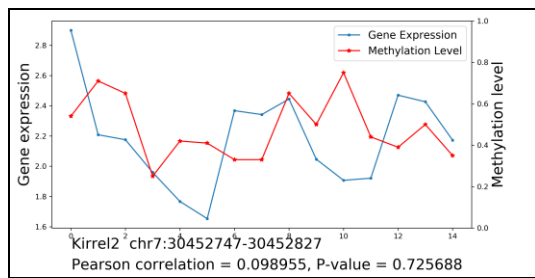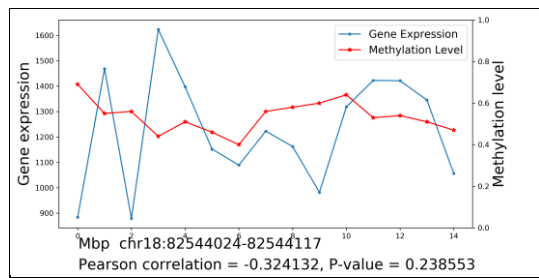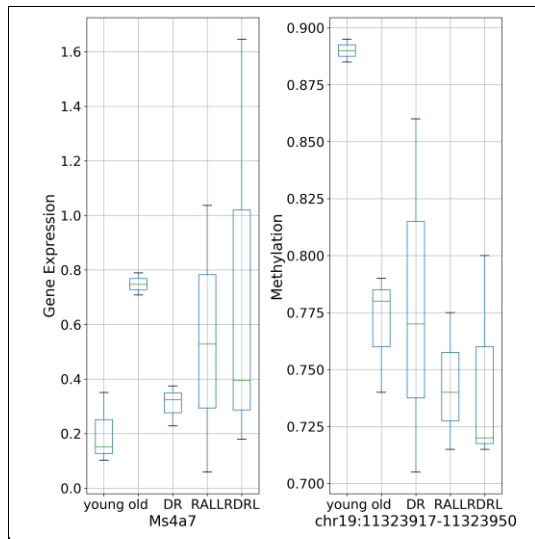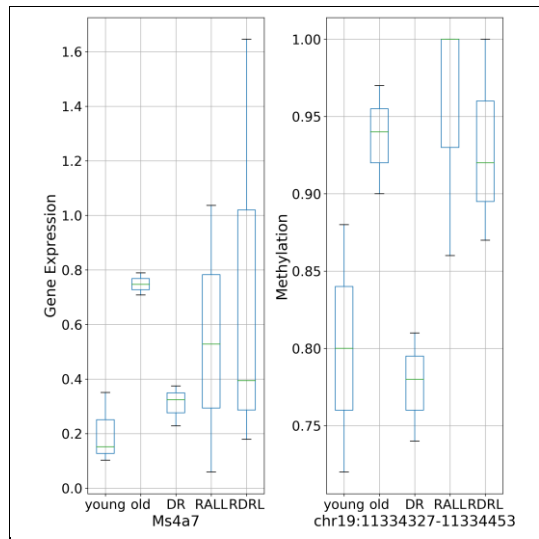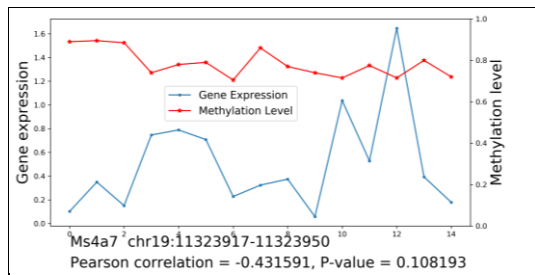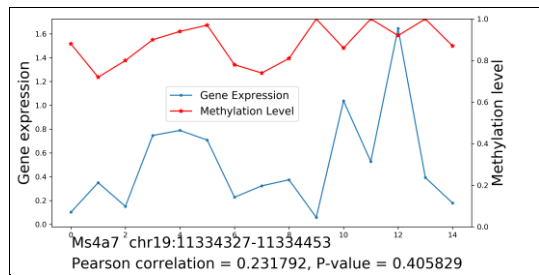

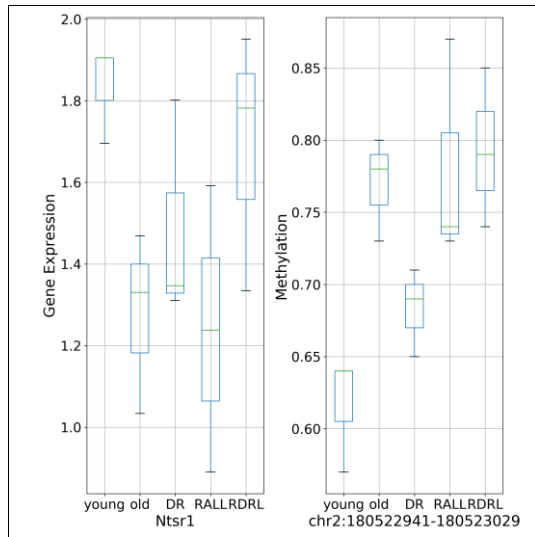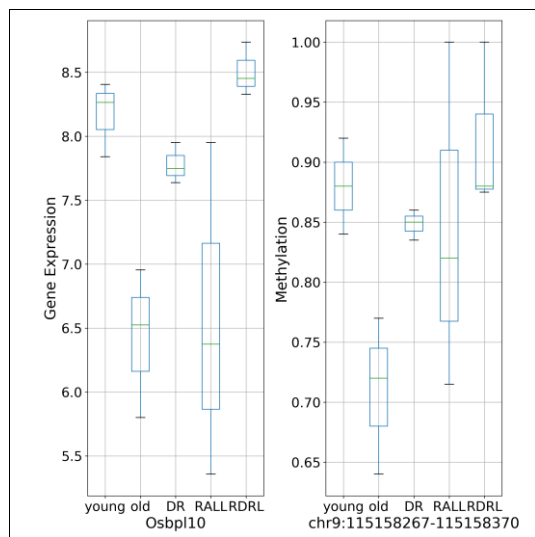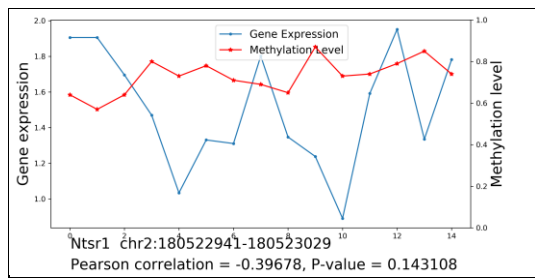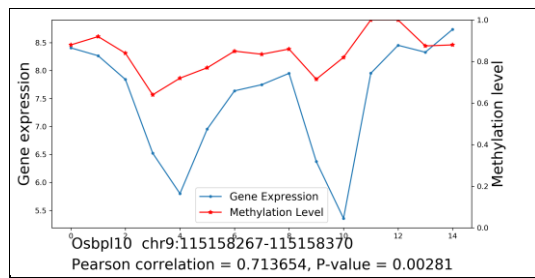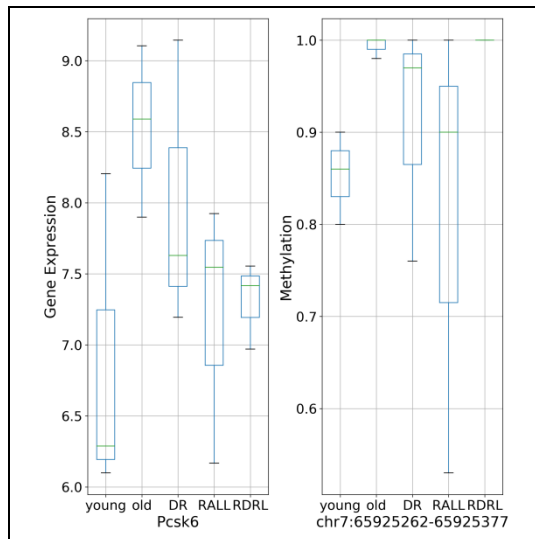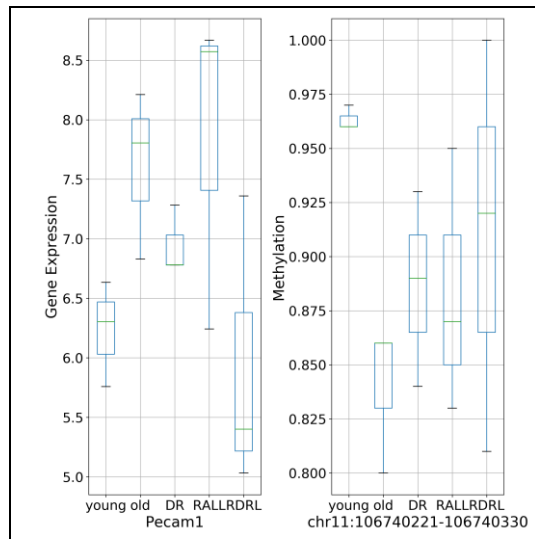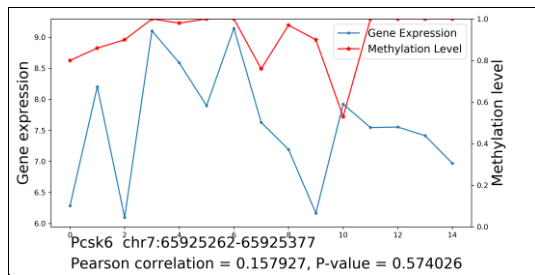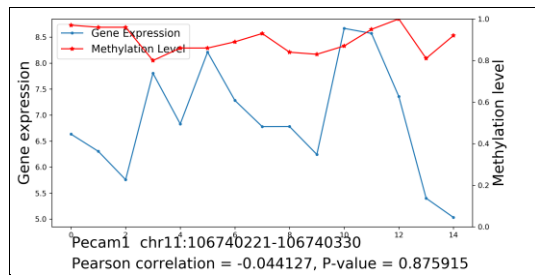

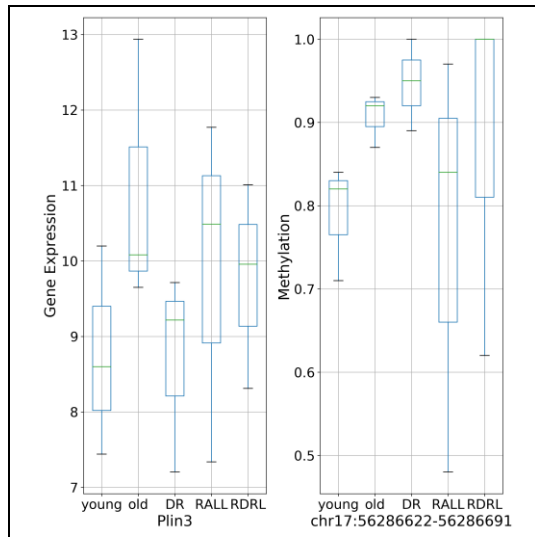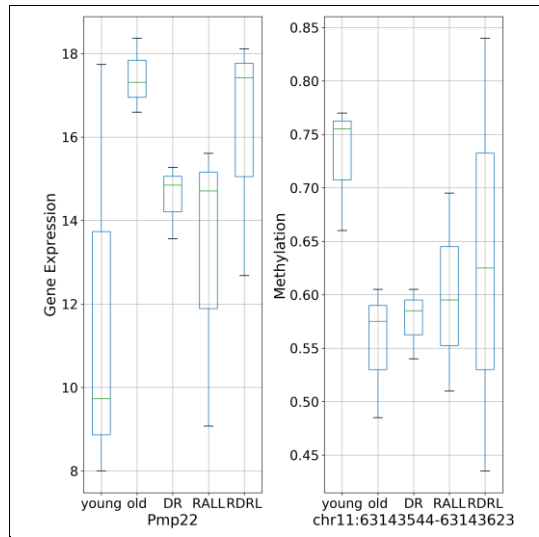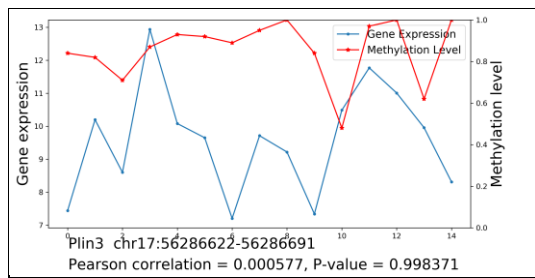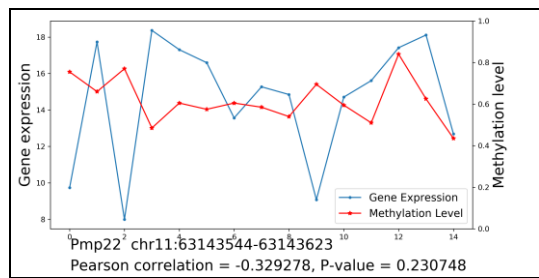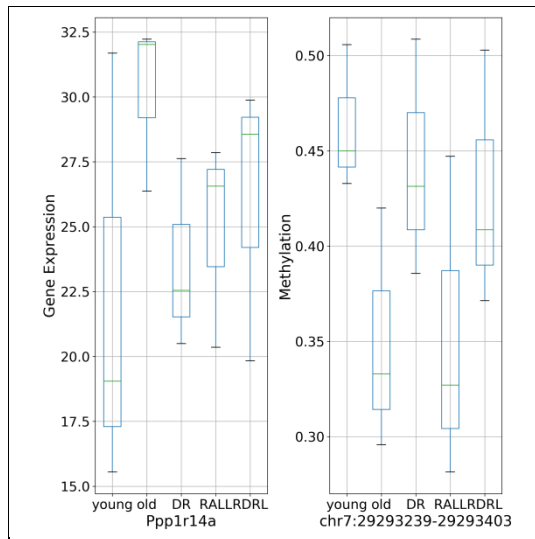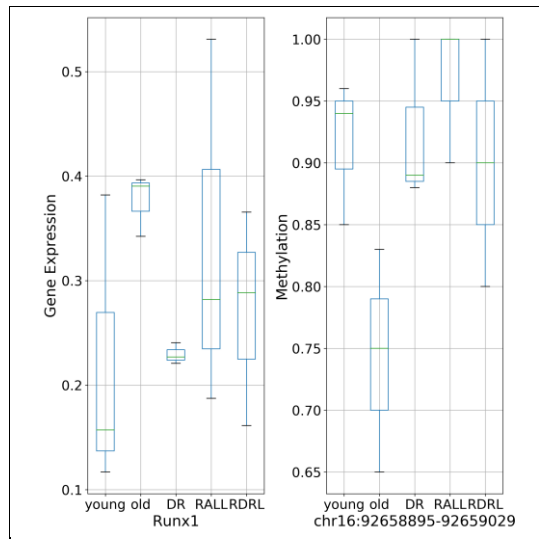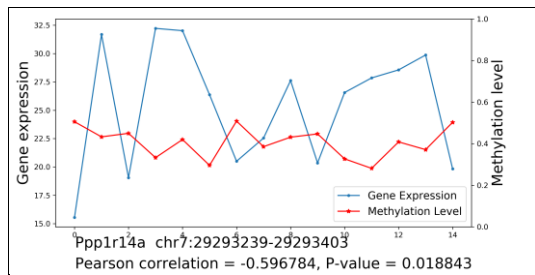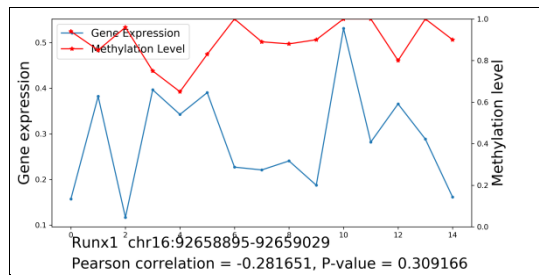

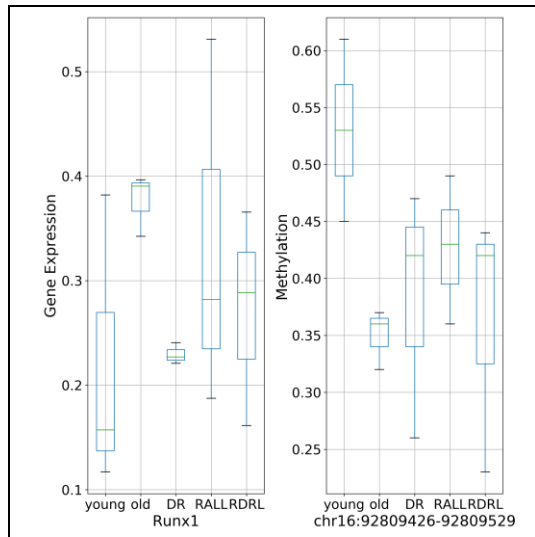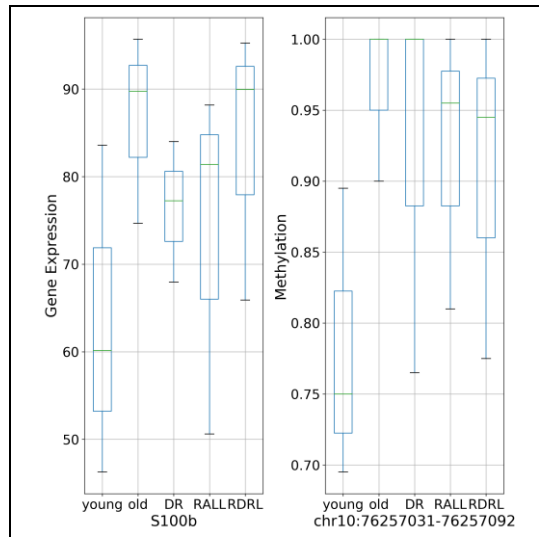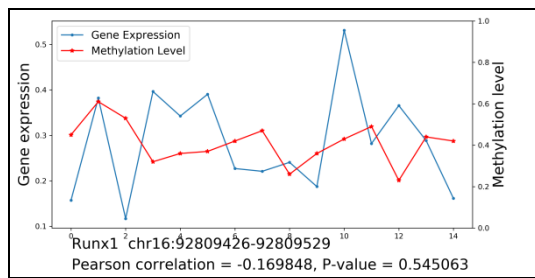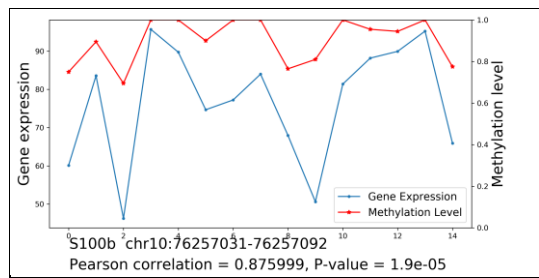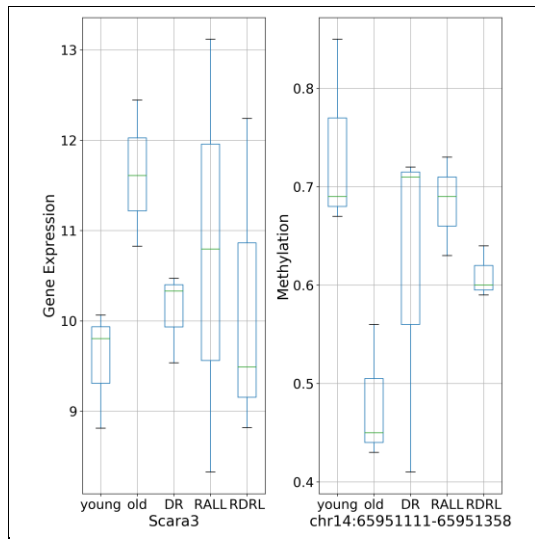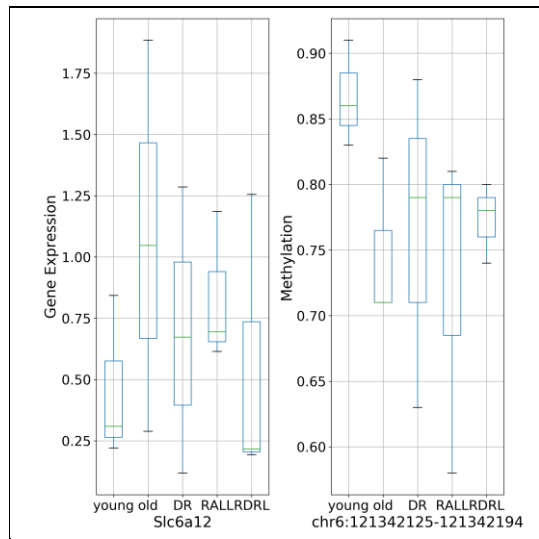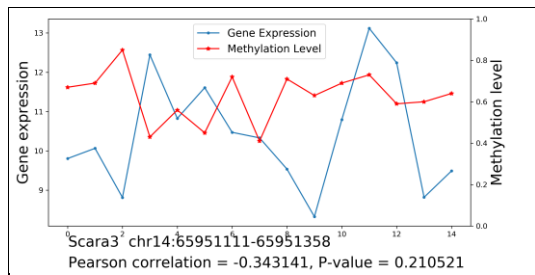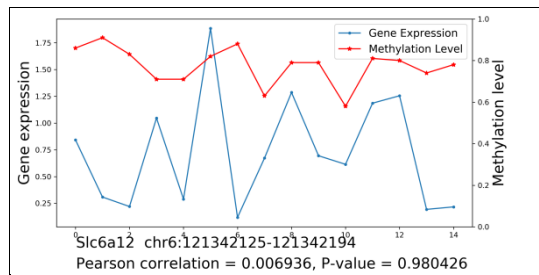

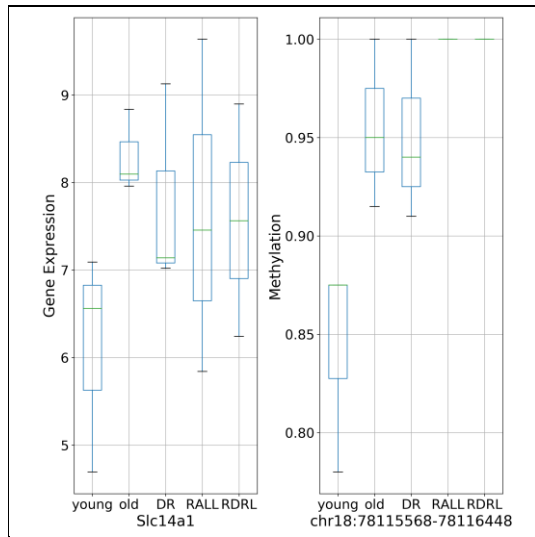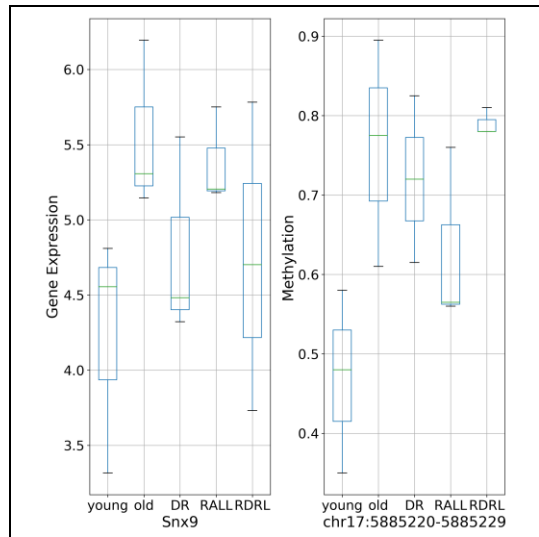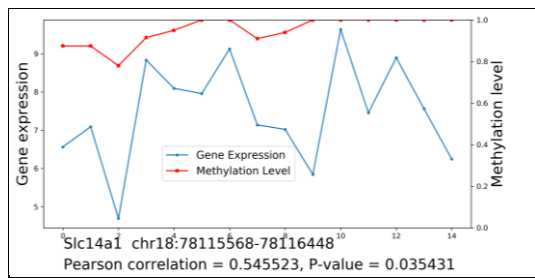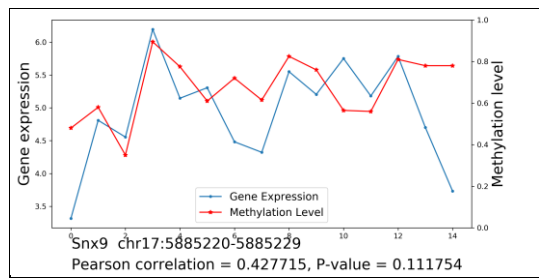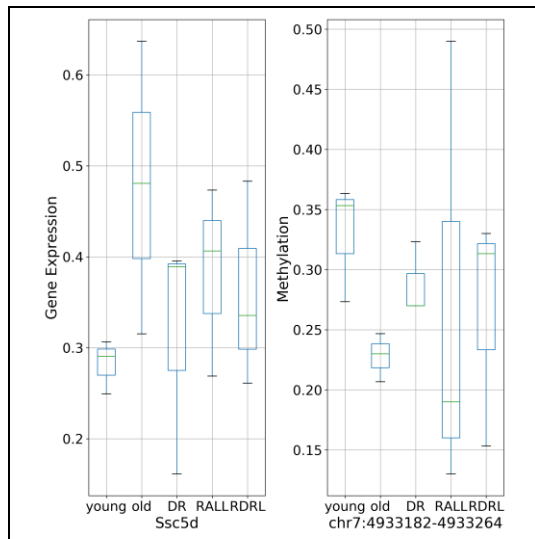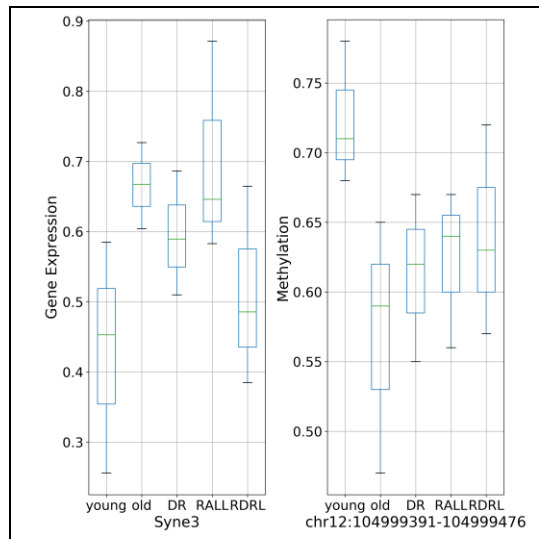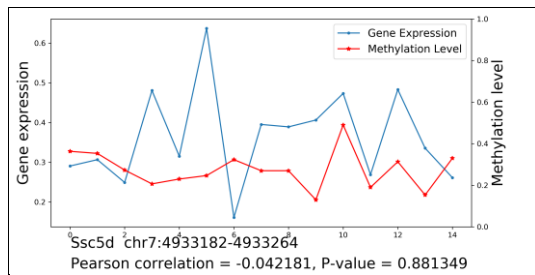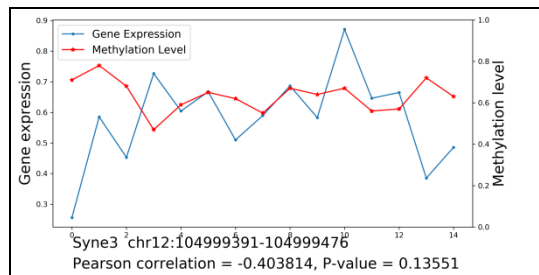

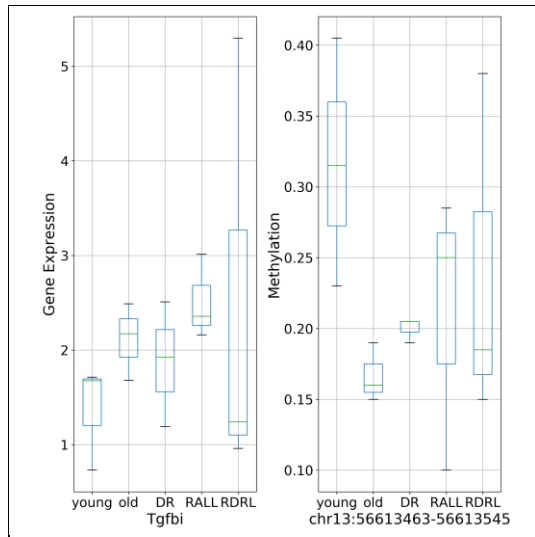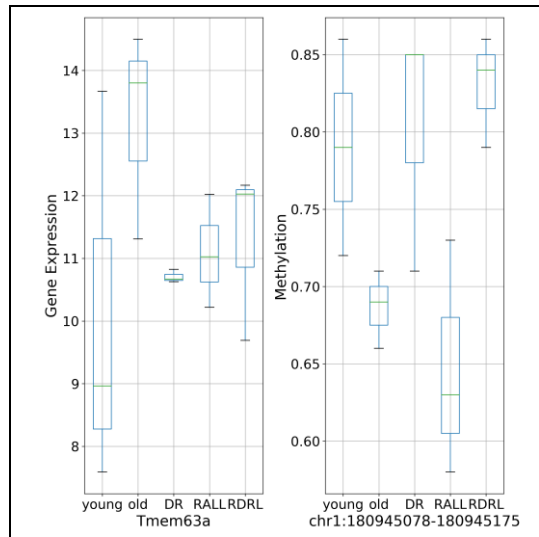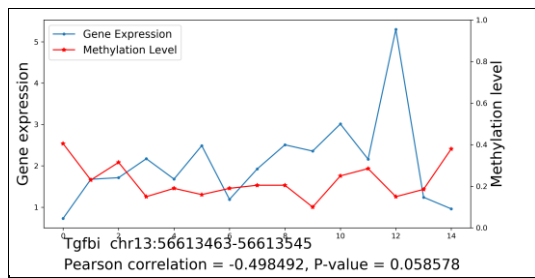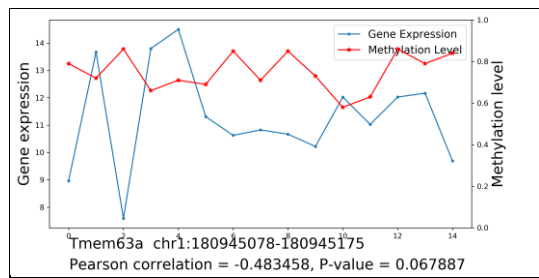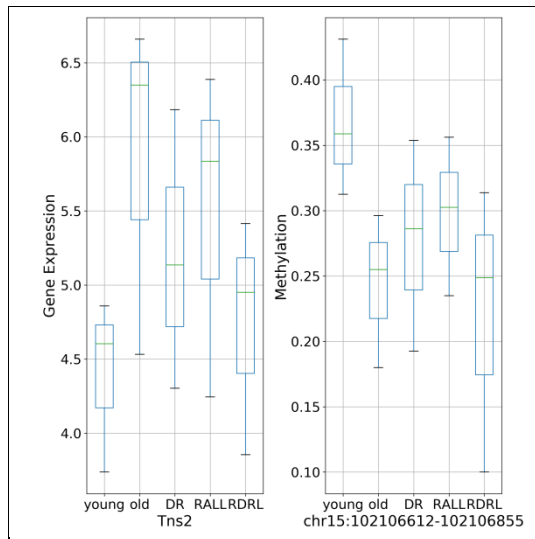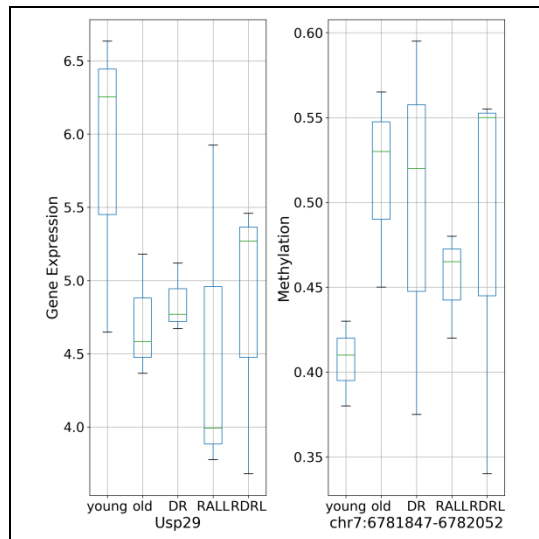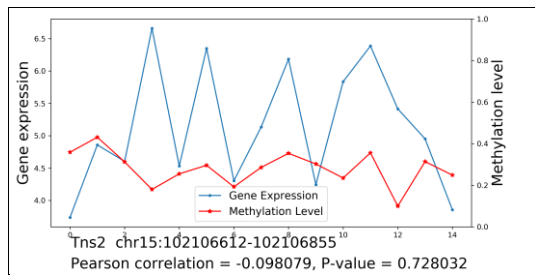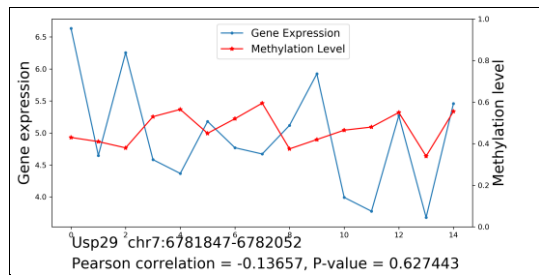

Supplement: Supplementary file 1 [file genes-13-00699-s001.zip › Figure S2 - Figures of Gene Expression and DMR methylation.pdf]
